# Propeller Fault Classification for Unmanned Aerial Vehicles and Explainable Artificial Intelligence-Based Feature–Model Matching

**DOI:** 10.3390/s26185845

**Published:** 2026-09-15

**Authors:** Ahmet Çağdaş Seçkin

**Affiliations:** Department of Computer Engineering, Faculty of Engineering, Aydın Adnan Menderes University, 09100 Aydın, Türkiye; acseckin@adu.edu.tr or seckin.ac@gmail.com

**Keywords:** unmanned aerial vehicle, propeller fault diagnosis, multisensor fusion, ensemble learning, explainable artificial intelligence, data leakage, vibration and acoustic analysis

## Abstract

The spread of unmanned aerial vehicles in daily operations makes the early and reliable diagnosis of propeller faults necessary. However, the performance values reported for such systems are usually obtained with sample level splits, and it is not known which feature representation should be matched with which learner. In this study, a leakage-free feature–model matching framework is presented for propeller fault classification. Microphone and six-axis inertial measurement unit data have been collected on a test bench with 980 kV and 1400 kV motors for one healthy and eight faulty propeller conditions at 16 throttle levels, and 8490 windows of 1 s have been extracted from 1735 measurement files. Four scalar feature sets and three time-frequency representations have been matched with seven ensemble learners and three compact convolutional networks under a file atomic split, and the permutation ranking of the best model has been returned to the feature selection stage. The highest macro-F1 value of 0.8027 and an accuracy of 0.8816 have been obtained with the stacked ensemble trained on the 52 input subset ranked by explainability. It is seen that the time domain statistics and the accelerometer axes are dominant, that three inertial axes reach a macro-F1 of 0.7661, and that cepstral and envelope features stay below the Welch-based features at the sampling rate of 90.9 Hz. The cross-motor experiments have shown that the models depend strongly on the motor class, and the McNemar test has confirmed that the difference between the ensemble branch and the compact convolutional branch is not accidental. In this way, the framework can be used as an evaluation protocol for low-cost multisensor setups on low-level devices.

## 1. Introduction

Unmanned aerial vehicles are widely used in many fields such as agriculture, infrastructure inspection, logistics, search and rescue and environmental monitoring [1,2,3,4,5]. Because these platforms work close to people and equipment, their reliability and maintenance processes must be handled as carefully as in classical aviation applications. Predictive maintenance approaches have been shown to reduce unplanned downtime and to improve service quality in the aviation sector, but the performance of data-driven models can be limited by the imbalance of the class distribution and by the small number of rare fault samples [6]. The same limitations are also valid for rotary-wing unmanned aerial vehicles.

Fault detection and isolation comprise a basic component of flight safety in an unmanned aerial vehicle. The comprehensive review by Puchalski and Giernacki classifies the methods in this field as model-based, signal-based and data-driven approaches, and it underlines that propeller faults are the most frequent actuator faults [7,8,9]. Because the propeller is the component where thrust is produced directly, even relatively small defects such as a crack, a notch, wear or warping lead to aerodynamic imbalance, to increased vibration, to higher energy consumption and to a loss of flight stability [10]. Fault-tolerant control architectures have been proposed so that the vehicle can land in a controlled way when one or two propellers fail completely, but these architectures can only be triggered by a fast and accurate diagnostic layer [11].

When the studies in this field are examined, it is seen that the existing approaches can be grouped according to three basic principles. The first group consists of model-based methods, which compare the measured response with the output of an analytical or identified dynamic model of the rotor and detect the fault from the residual [7,12]. These methods provide a physical interpretation and need very few training data, but they depend on an accurate model and lose their sensitivity when the model parameters drift with wear, temperature and payload. The second group consists of signal-based methods, which extract a known indicator such as the vibration amplitude, the imbalance magnitude, the harmonics-to-noise ratio or a band energy from the measured signal and apply a threshold or a rule [10,13,14,15,16]. These methods are simple and can run on low-level devices, but their thresholds have to be tuned for each platform, and they mostly answer the binary question of whether a fault exists. The third group consists of data-driven methods, which learn the mapping from hand-designed features or from raw representations to the fault class with machine learning or deep learning [17,18,19,20,21,22,23]. These methods can separate many fault types and severities, but they assume that the training and test data come from the same distribution, and their reported performance depends strongly on how the data are split. The present study belongs to the third group, and it is designed so that the last two limitations, namely the split protocol and the distribution shift between hardware, are measured openly instead of being assumed away.

The progress of data-driven diagnostic methods is directly related to the existence of publicly available measurement sets. The ALFA dataset, which contains motor and control surface faults on a fixed-wing platform, has prepared the ground for fault detection studies with real flight data [8]. On the rotary-wing side, the PADRE repository presents different rotor fault configurations with measurements collected from four accelerometers and four gyroscopes [24], and its extended version defines new quality indicators so that classifiers can be compared [9]. Real-time classification experiments on the same repository have shown that the processing time and the accuracy depend strongly on the parameter selection [25]. On the acoustic side, a dataset collected with a microphone array placed on the vehicle, which covers all the broken propeller configurations and contains more than five hours of recording, has reported 98.30 percent accuracy with a transformer encoder architecture [17].

Vibration-based methods form the most common approach because an inertial measurement unit is available on almost every platform. Ghalamchi and Mueller have proposed a method that estimates the mass magnitude of the propeller imbalance by relating the accelerometer output to the motor commands and that needs a low computational load [13,14]. Zhang et al. have developed a frequency estimation approach that works in the time domain for the diagnosis of blade damage [15]. Baldini et al. have combined finite impulse response filters with sparse classifiers by using only the inertial measurement unit data available on board, and they have reported 93.37 percent accuracy with a binary decision tree and 98.21 percent accuracy with a linear support vector machine [18]. Sonmezocak has detected not only propeller damage but also the loosening of the rotor and arm screws with micro-electromechanical vibration measurements, and obtained a maximum accuracy of 99.40 percent in the determination of the damage severity [19]. Al-Haddad and Jaber have increased the accuracy from 92.52 percent to 98.85 percent by giving the time-domain features selected with the ReliefF ranking method to a support vector machine [20].

Acoustic-based methods use the change in the sound signature produced by a damaged propeller. Cinoğlu has extracted the harmonics-to-noise ratio feature from sound recordings taken at three different thrust levels and has obtained 81.24 percent accuracy and an 83.76 percent F1 score with a Gaussian naive Bayes classifier [16]. Yi et al. have built a realistic dataset by mixing sound recordings collected from three different vehicles in an anechoic room with campus noise, and they have shown that a multitask network which uses direction classification as an auxiliary task outperforms single-task models [26]. Liu et al. have proposed a risky-flight detection framework that uses only the propeller rotation sound and that contains transfer learning and Bayesian optimization [27]. Berghout and Benbouzid have reported that representation learning based on recurrent expansion reduces concept drift on acoustic emission data [28].

The limits of approaches that rely on a single modality have brought multisensor fusion studies forward. Tsai et al. have proposed a convolutional network that trains the current and hydrophone signals separately and merges them in the last layer, and they have reported 99.94 percent accuracy in a pool environment and 99.06 percent accuracy in a lake environment [21]. Hadi et al. have classified pre-flight abnormalities with an average accuracy of 97.5 percent by using contactless radio frequency vibration sensing [5]. These studies show that different physical quantities carry complementary information, but in most cases, it is not quantified in an open way which modality is decisive for which fault type.

Deep learning architectures can learn discriminative patterns directly from the raw signal or from the time-frequency representation instead of using hand-designed features. Yang et al. have reported a diagnostic accuracy above 98 percent in a noisy environment with a deep residual shrinkage network [22]. Wang et al. have proposed a local and global attention-based graph neural network by treating propeller fault diagnosis as a node classification problem in a non-Euclidean space [23]. A dual-architecture convolutional network based on the divide and conquer principle has been developed for the simulation to reality transfer problem [29], and machine learning approaches supported by a hybrid data generation model have been proposed [30]. Brulin et al. have defined a method for producing a fault database by using simulation and experimental data together [31].

In recent years, fault diagnosis methods based on large language models have become a new research direction in the mechanical fault diagnosis field. In these methods, the vibration signal or the features extracted from it are converted into a text or token sequence, and a pretrained language model is fine-tuned so that the fault class and a textual explanation are produced together [32]. An adaptive industrial large language model has been proposed for mechanical fault diagnosis under variable operating conditions, and it has been reported that the semantic representation carried by the pretrained model reduces the loss of performance when the speed and the load change [33]. The recent review of Nie et al. places these methods as a new paradigm that combines data-driven learning with knowledge guidance, and it underlines that the variable operating condition problem is one of the main motivations of this paradigm [34]. It is thought that this direction is promising for the operating condition transfer problem that is also observed in this study. However, these models contain hundreds of millions of parameters and need graphics processing units at inference time, so they are not yet suitable for the flight computer of a small unmanned aerial vehicle. For this reason, in this study, compact learners that can run on low-level devices have been preferred, and the large language model-based approaches are discussed as a direction for the cross hardware transfer problem in Section 3.10.

Determining not only the presence of the propeller damage but also its type and its severity is a target that has come forward in recent years. Pose et al. first developed a neural network-based damage detection method [35], and afterwards, they proposed a combined model that estimates the damage type, the severity and the affected rotor together by using only inertial measurements and control commands [36]. Zou et al. have placed fault detection on a physical basis by building the analytical model of the propeller dynamics [12]. These studies show that multiclass and severity-aware diagnosis is clearly harder than the single-class detection problem.

The basic principle, the main assumption, the advantages and the limitations of these groups are summarized in Table 1. Accordingly, the vibration-based methods assume that the inertial sensor is rigidly coupled to the rotor and that the imbalance forcing is observable in the band of the sensor, the acoustic methods assume that the background noise of the environment is stationary, and the deep learning methods assume that a large number of independent samples are available. When the studies in Table 1 are examined, it turns out that three questions stay open in most of them. The first one is that the split protocol is not stated or is applied randomly at the sample level, so the reported values may contain information leakage between overlapping windows of the same recording. The second one is that the contribution of each sensor and each feature domain is not quantified, so it is not known which representation should be matched with which learner. The third one is that the transfer between different motor and propeller hardware is rarely tested. The goal of this study is to answer these three questions on a single multisensor dataset with a fixed evaluation protocol, and the objectives are the quantification of the leakage-free performance, the identification of the best feature and model pair together with the reasons behind it, and the measurement of the transfer between two motor classes.

Our previous study has shown that microphone, accelerometer, gyroscope and temperature data collected on a test bench with a 980 kV motor can be converted into time-frequency images with the short-time Fourier transform, and that five deep learning architectures can separate nine propeller conditions with high accuracy [37]. That study is a proof of concept which shows that multisensor fusion works in propeller diagnosis, and it forms the starting point of this paper. In order to move from a proof of concept to a field application, three questions have to be answered. The first one is whether the obtained performance is valid between different parts of the same measurement session or on measurement files that have never been seen before. The second one is which sensor, which feature domain and which operating variable the performance comes from. The third is how far the same model can be transferred to a different motor class. This paper is built on the previous study by extending the same experimental setup and by designing the evaluation protocol so that these three questions can be answered.

This study has been designed as the direct continuation of the mentioned earlier work [37], and it extends that work in four points. First, the dataset has been extended to 1735 measurement files so that it covers a second motor class and 16 separate throttle levels. Second, a stratified 80/10/10 split that treats the physical measurement file as indivisible has been applied, and the leakage audit has been reported as a separate table. Third, a scalar statistics branch and a raw time-frequency branch have been designed together on the same windows, and a total of 33 pairs formed by four feature sets with six ensemble methods and three time-frequency representations with three convolutional networks have been evaluated in a single grid. Fourth, it has been quantified with permutation importance, SHAP and integrated gradient methods which sensor, which domain and which representation contributes to the decision, and the robustness of the results has been tested with sensor ablation, inertial axis subset ablation, frequency representation ablation, operating input ablation, cross-motor generalization, repeated splits and the McNemar test. The convolutional architectures that have been used are compact.

It is useful to highlight the innovations and contributions of the proposed feature–model matching framework compared to the existing methods:

- The matching of the feature representation with the learner is treated as a design variable and not as a fixed choice. Four scalar feature sets and three time-frequency representations are matched with six ensemble methods and three convolutional networks in a single grid under one split, one prior correction and one metric set, so the effect of the representation and the effect of the learner can be separated.
- The explainability analysis is placed inside the pipeline as a feedback step instead of being reported only as a post hoc illustration. The permutation importance ranking obtained on the full feature set is returned to the feature extraction stage, a reduced subset of 52 inputs is built from it, and this subset is evaluated in the same grid as the other sets.
- A file atomic stratified split with a written leakage audit is used, and the drop from the window-level split of the previous study to the file-level split is quantified on the same test bench.
- The dependence of the results on the sensor set, on the operating inputs, on the motor hardware and on the random seed is measured with six ablation and robustness experiments, and the difference between the branches is verified with a paired McNemar test.

The novelty of the framework is therefore not a new learner or a new transform, but the systematic and leakage-free procedure with which an existing feature family is matched with an existing learner family and the reasons behind the resulting pair are made explicit. The article is organized as follows. Section 2 states the problem and describes the experimental setup, the feature extraction, the models and the evaluation protocol. Section 3 presents the results, the explainability analyses and the ablation experiments. The article is finalized with the conclusion section, where the limitations and the planned studies are given.

## 2. Materials and Methods

**Figure 1:**
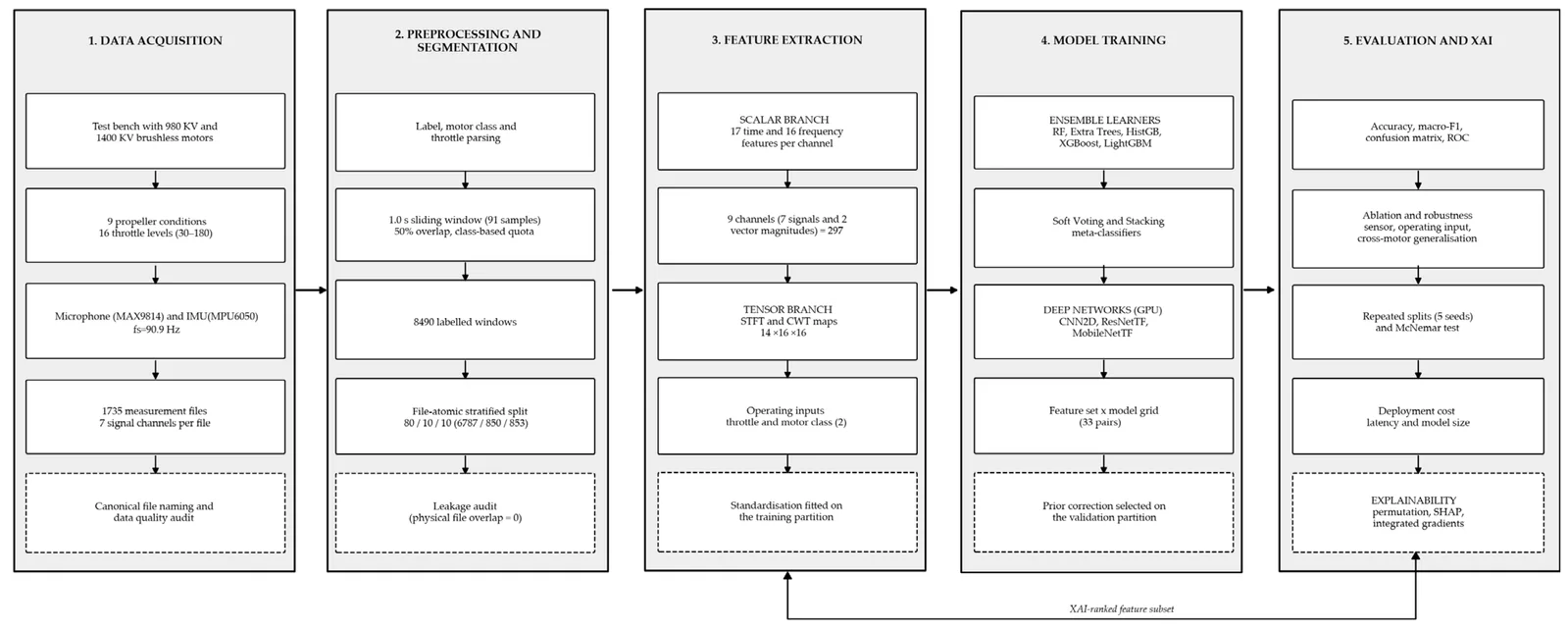
Overall methodology of the proposed propeller fault classification pipeline.

The whole study has been designed as a five-stage pipeline that starts from the raw measurement files and reaches the explainability and deployment cost analyses. Figure 1 summarizes this flow as a block diagram. The first stage covers data acquisition, the second stage covers preprocessing and segmentation, the third stage covers feature extraction, the fourth stage covers model training, and the fifth stage covers the evaluation and explainability analyses. The boxes with dashed frames show the audit and verification steps applied at each stage. The dashed feedback path at the bottom of the diagram indicates that the permutation importance ranking obtained in the fifth stage is returned to the third stage and is used for building the reduced feature subset. The whole pipeline is run with a fixed random seed, and each stage produces its own tables and figures.

Before the stages are described, the problem addressed in this study is stated in an open way. What is given is a set of measurement files, each of which contains one microphone channel and six inertial channels sampled at 90.9 Hz on a test bench, together with the throttle command and the motor class of the file. What is required is the assignment of one of nine propeller conditions to each 1 s window of a file that has never been seen during training. The problem is therefore a nine-class supervised classification problem in which the sample unit is the window and the independence unit is the measurement file. The following conditions and assumptions are valid. The throttle command is constant inside a file, so the rotation speed is assumed to be approximately constant inside a 1 s window. The rotation speed itself is not measured, and only the throttle command and the motor class are known. The sampling rate is expected to be below the shaft frequency of the propeller for a large part of the throttle range, so the models are not expected to resolve the shaft orders, and they have to work with the low-frequency and aliased content of the rotating forcing. The training and test files come from the same bench and the same two motors, and the transfer to a different motor is measured separately in the cross-motor experiment. Under these conditions, the classifier is expected to operate as an offline or near real-time diagnostic layer with a latency below one millisecond per window on a low-level device.

The two branches of the pipeline are the answer to a single design question, namely which representation should be matched with which learner under these conditions. The scalar branch is designed for the case where the computational budget is small and the number of files is limited, so hand-designed statistics are matched with tree ensembles. The tensor branch is designed for the case where the discriminative pattern is expected to be local in the time–frequency plane, so raw maps are matched with compact convolutional networks. The two branches are not compared as two learners on the same input, but as two complete representation and learner pairs on the same windows, the same split and the same metrics. For this reason, the comparison in Section 3 is a comparison at the pipeline level, and it should not be read as a one-to-one comparison between a tree ensemble and a convolutional network. The TOPK_XAI subset does not move from the tensor representation back to the scalar representation. It is a feedback step inside the scalar branch, in which the permutation ranking of the best ensemble model trained on all 299 inputs is used to reduce the same feature set to 52 inputs, as shown by the dashed path in Figure 1. In this way, the study first selects the better branch, then reduces the input of that branch with its own explainability result, and finally tests the robustness of the selected pair.

### 2.1. Experimental Setup and Dataset

The measurements have been taken in a controlled setup where brushless direct-current motors are mounted on a test bench. Two different motor classes of 980 kV and 1400 kV have been used in the setup, each motor has been driven with a 20 A electronic speed controller, and the throttle command has been given through a microcontroller (Yinyan Model Tech. Ltd., Shenzhen, China). The acoustic data have been collected with a MAX9814 microphone module with automatic gain control, and the vibration and rotation data have been collected with an MPU6050 inertial measurement unit that contains a three-axis accelerometer and a three-axis gyroscope. The raw recordings have been stored as delimited text files. The mean sampling rate computed from the timestamps has been measured as 90.9 Hz and has been assumed constant throughout the pipeline.

The reason why the microphone is used together with the inertial measurement unit is as follows. The inertial sensor is coupled to the motor mount and observes the mechanical imbalance forcing, while the microphone is a contactless sensor and observes the aerodynamic effect of the blade damage on the flow, which does not need to produce a mass imbalance. A scratch or surface wear changes the pressure field around the blade before it changes the mass distribution, so it is expected that the acoustic channel carries a component that is not present in the inertial channels. In addition, the microphone module costs less than one dollar and does not need any mechanical installation on the rotor. Whether this expectation holds is not assumed but measured with the sensor ablation in Section 3.7, where the contribution of the microphone alone and as an addition to the inertial channels is quantified.

**Figure 2:**
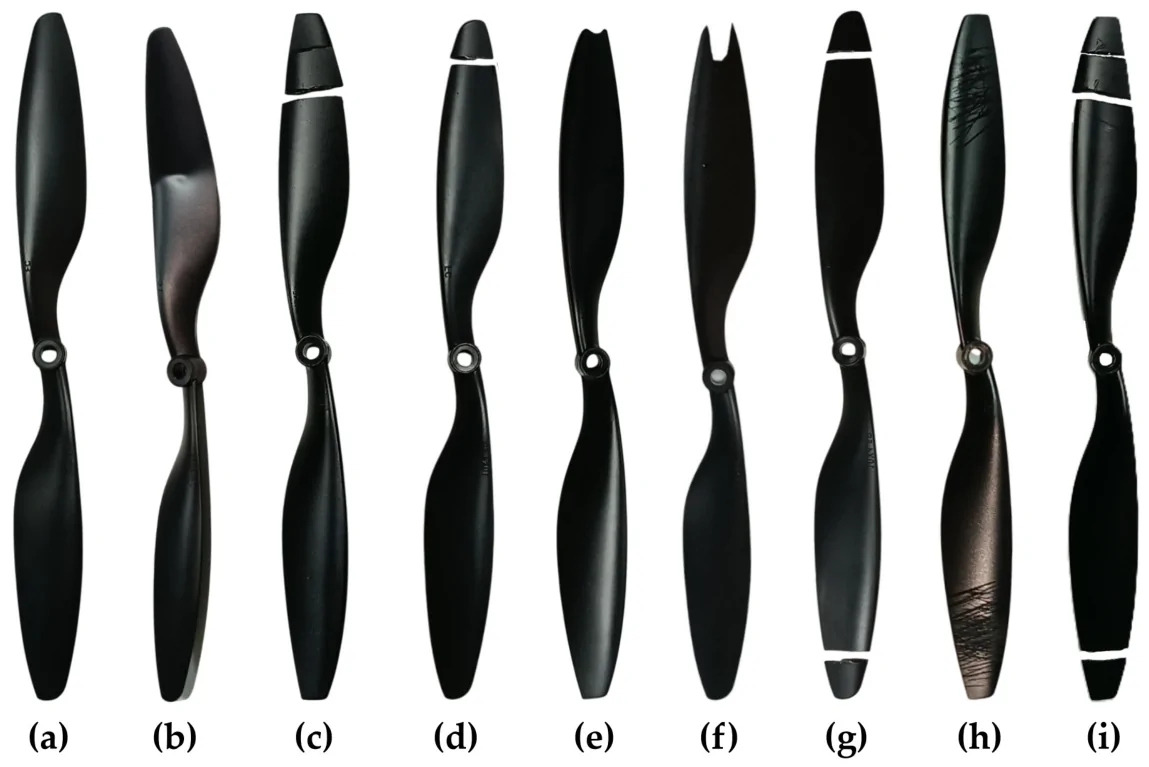
Healthy propeller and the induced fault conditions used in the experiments. (**a**) healthy; (**b**) warped; (**c**) single cut long; (**d**) single cut short; (**e**) notch short; (**f**) notch long; (**g**) two cut short; (**h**) scratch; (**i**) two cut long.

The propeller conditions are exactly the same as the definitions in the previous study [37]. The healthy condition indicates the reference propeller that carries no mechanical deformation and no surface wear. The long-notch and short-notch conditions represent the beginning of a fatigue crack caused by cyclic loading, the warped condition represents ageing effects such as ultraviolet exposure and thermal cycling, and the scratch condition represents the surface damage that occurs after contact with airborne particles. The single long-cut and single short-cut conditions model a linear cut on a single blade, and the two long-cut and two short-cut conditions model two separate fracture regions on the same propeller. All the damages have been produced with repeatable methods on replaceable propeller modules. Figure 2 shows the healthy and the damaged propeller samples.

For each propeller condition, the throttle command has been changed between 30 and 180 in steps of 10, and 16 different operating points have been scanned in this way. The names of the measurement files have been converted into a canonical form that consists of the measurement identifier, the fault label, the motor class and the throttle level fields. Two kinds of naming anomaly caused by the raw recording software have been repaired at this stage. The first one is the label spelling errors, and these have been removed with a normalization dictionary. The second one is the wrong codes that occur when the repetition counter is added in front of the throttle field, and these have been corrected only when the remaining suffix corresponds to a valid throttle level. The renaming operation is written into a separate log file so that it can be audited. In addition, the time, speed and temperature columns have been removed from the raw files. Temperature is not used as a model input because this quantity drifts in one direction during a measurement session and therefore encodes the identity of the measurement file rather than the physical condition of the propeller.

Table 2 gives the number of measurement files per class, the number of windows obtained after segmentation and the motor classes in which the class has been observed. Of the 1735 measurement files, 1302 belong to the 980 kV motor, and 433 belong to the 1400 kV motor. The healthy class forms about 57 percent of the dataset with 989 files, while the faulty classes vary between 50 and 130 files. This imbalance reflects real maintenance scenarios, but it has also been handled separately during model training.

### 2.2. Segmentation and Class Balancing

Each measurement file has been divided into sliding windows of 1.0 s length. At the measured sampling rate, this length corresponds to 91 samples, and an overlap of 50 percent has been used between the windows. In order to prevent the healthy class from overwhelming the other classes, the step size has been adapted per class. The target total number of windows for each class has been divided by the number of files of that class in order to set a quota per file, and the starting points inside a file have been selected at equal intervals according to this quota. In this way, only real overlapping windows have been used without generating synthetic data. As a result, 8490 labelled windows have been obtained, and 4.89 windows fall on each file on average.

### 2.3. Training, Validation and Test Split with the Leakage Audit

A defect that is met often in the fault diagnosis literature is the random splitting of overlapping windows. The formal analysis of data leakage made by Kaufman et al. shows that, in cases where the independent and identically distributed assumption is broken, splits made over the sample unit instead of the observation unit carry information about the target variable that is not legitimate [38]. When two windows produced from the same raw recording and sharing mostly the same signal segment are placed one in training and the other in testing, the model is rewarded for its memorization ability instead of real generalization. In this study, the physical measurement file has been treated as indivisible. The stratified split has been carried out over unique files and not over windows, a test set of 10 percent has been separated first, and a second stratified split has been applied on the remaining part so that a validation set of 10 percent is obtained. After the split, it has been verified that the file intersections between the three partitions are empty.

Table 3 gives the number of windows and files of the partitions, their ratios and the class distribution. In Table 3, the class abbreviations are used as follows: HE: healthy, NL: notched long, NS: notched short, SC: scratched, SL: single-cut long, SS: single-cut short, TL: double-cut long, TS: double-cut short, WA: twisted. The training partition contains 6787 windows and 1387 files, the validation partition contains 850 windows and 174 files, and the test partition contains 853 windows and 174 files. The fact that the window ratios and the file ratios are very close to each other shows that the number of windows per file is distributed in a balanced way between the classes. The class columns confirm that all nine classes are represented in every partition. The class imbalance has been removed with random oversampling only over the training indices. The natural distribution of the validation and test partitions has not been touched, because these partitions have to reflect the real operating conditions. In addition, a prior correction coefficient has been defined in order to reduce the pull of the training class prior towards the healthy class. This coefficient has been selected only so that the macro-F1 value on the validation partition is the largest, and the test labels have not been used at any stage.

### 2.4. Feature Extraction

Two parallel representations have been produced for each window. The scalar branch treats the accelerometer and gyroscope vector magnitudes as channels in addition to the seven signals that consist of the microphone and six inertial axes. The vector magnitudes are computed as the square root of the sum of the squares of the axis components, and they give a vibration measure that is relatively stable against the orientation changes of the platform. In this way, the total number of channels rises to nine.

For each channel, 17 time-domain and 16 frequency-domain features have been computed. The time-domain features consist of the mean, the standard deviation, the minimum and maximum value, the median, the interquartile range, the median absolute deviation, the root mean square, the peak-to-peak amplitude, the skewness, the kurtosis, the energy, the zero crossing rate, the crest factor, the shape factor, the impulse factor and the linear slope. The frequency-domain features have been derived from the power spectral density estimated with the Welch method, and they contain the dominant frequency, the spectral centroid, the spectral bandwidth, the normalized spectral entropy, the total power, five band energy ratios and the first six normalized amplitudes taken from the fast Fourier transform with a Hanning window. The band limits have been divided into five slices between 0 and 45.5 Hz, and the upper limit corresponds to the Nyquist frequency.

The choice of the Welch estimate and the treatment of the rotating nature of the system need to be justified. The propeller is a rotating source, and the forcing it produces is periodic per revolution, so the vibration response is expected to be cyclostationary rather than strictly stationary. However, three conditions of the present dataset limit the representations that can be applied. The first one is that the rotation speed is not measured and only the throttle command is known, so order tracking and angular resampling, which need an angular reference, cannot be applied to the recordings. The second one is that the sampling rate of 90.9 Hz gives a Nyquist frequency of 45.5 Hz, which is expected to be below the shaft frequency for a large part of the throttle range. For this reason, the harmonic family of the shaft order is not resolved in the spectrum, and it appears in an aliased form, so cepstral analysis, which detects the frequency of a harmonic family, and cyclic spectral analysis, which needs the cyclic frequency to be observable, cannot be interpreted physically on these recordings. A cepstral and an envelope spectrum feature set have nevertheless been computed as a methodological check, and they are compared with the Welch-based set in Section 3.8. The third one is that a window contains 91 samples. With a segment length of 64 samples and an overlap of 32 samples, the Welch estimate reduces to a single Hann-windowed periodogram with a resolution of about 1.4 Hz, so no averaging over segments takes place, and the loss of information that is attributed to the averaging of periodograms does not occur here. The stationarity inside the window has been checked with four tests on a sample of the windows, and the results are given in Section 3.1 together with representative raw windows of the inertial channels. Because of these conditions, the frequency-domain features of this study should be read as low-resolution band descriptors of the aliased low-frequency content and not as an order spectrum. Accordingly, the comparison between the time domain and the frequency domain reported in Section 3 is valid for the specific Welch-based features used here, and a different frequency representation obtained at a higher sampling rate with an angular reference may change this ordering. The extension of the pipeline with an order-tracked, cepstral and cyclostationary representation requires a new measurement campaign and is stated as a separate study in Section 4.

When the 33 features per channel are multiplied by nine channels, 297 scalar features are obtained. Two operating inputs, which are the throttle level and the motor class, are added to these, and the total input dimension becomes 299. Providing the operating inputs to the model is a deliberate choice, because the same fault produces a different vibration signature at different throttle levels. The effect of this choice on the results has also been measured. Table 4 summarizes the composition of the feature vector.

The tensor branch produces a 14-channel time–frequency stack from the same window. The first seven channels are the logarithmic power spectrogram obtained from the short-time Fourier transform of each signal. The next seven channels are the continuous wavelet transform scalogram computed with the Morlet mother wavelet at scales from 1 to 16. Each map has been resampled to a size of 16 by 16 and has been normalized so that each channel has zero mean and unit variance. In this way, the input of the deep branch consists of a tensor of size 14 by 16 by 16 and a two-dimensional operating vector, and the total number of inputs is 3586. The normalization statistics have been computed only from the training partition.

### 2.5. Classification Models

Five base learners have been used in the ensemble branch. The random forest consists of 700 trees and evaluates as many candidates as the square root of the number of features at each split. The extra trees use the same number of trees but work with a feature ratio of 0.8. The histogram-based gradient boosting has been configured with 450 iterations, a learning rate of 0.06 and 31 leaf nodes. XGBoost uses 700 trees, a depth of 8, a learning rate of 0.05 and a subsampling ratio of 0.9. LightGBM has been trained with 700 trees, a learning rate of 0.04 and 31 leaves under balanced class weights. Two high-level combiners, which are soft voting and stacked generalization, have been built on these five learners. In the stacked generalization, the top-layer classifier is a logistic regression, and the base predictions are produced with three-fold cross validation.

Three compact convolutional networks have been defined in the deep branch. The common body consists of two three-by-three convolution layers, batch normalization, SiLU activation and maximum pooling, followed by a third convolution layer that doubles the width and an adaptive average pooling. In the classifier head, the pooled features are concatenated with the operating vector and are passed through a fully connected layer with 128 neurons and a dropout of 0.25. The three architectures differ only in their base widths. CNN2D uses a width of 32, ResNetTF uses 48, and MobileNetTF uses 24. The naming reflects the reference made to the residual connection [39] and lightweight embedded network [40] design philosophies, and the architectures are not exact copies of these references but compact versions adapted to the small maps of size 16 by 16. The training runs for at most 150 epochs with the AdamW optimizer, a learning rate of 0.001, a weight decay of 0.0001 and a batch size of 256, and early stopping is applied when the validation loss does not improve for 20 epochs. All the deep trainings have been carried out on a graphics processing unit.

### 2.6. Feature Set and Model Grid

A grid with two parts has been built in order to determine which representation should be matched with which learner. Four feature sets have been defined on the ensemble side. The TD set contains only the time-domain statistics, the FD set contains only the frequency-domain statistics and the TD + FD set contains both of them. The fourth set, which is TOPK_XAI, is defined in detail below. Two operating inputs are added to all four sets. These sets have been matched with six ensemble methods, which has produced 24 pairs. Three time–frequency subsets have been defined on the deep side. STFT uses only the spectrogram channels, CWT uses only the scalogram channels, and STFT + CWT uses all 14 channels. These three sets have been matched with three architectures, which has given nine pairs. A total of 33 pairs have been evaluated under the same split, the same prior correction and the same metrics. The best pair has been selected first according to the macro-F1 metric and then according to the accuracy.

#### Construction of the TOPK_XAI Feature Subset

TOPK_XAI is a feature subset that is selected according to the decision behavior of the model itself and not according to a predefined statistical family. The construction procedure consists of five steps. In the first step, the most successful ensemble model trained on all 299 inputs of the TD + FD set is taken as the reference. In the second step, the baseline accuracy of this model on a subsample of 800 windows taken from the test partition is computed. In the third step, each of the 299 inputs is shuffled randomly in turn and alone, the model is evaluated again on the same subsample without being retrained, and the drop that occurs with respect to the baseline accuracy is recorded. The shuffling is repeated three times for each feature and the mean of the drops is accepted as the importance score of that feature. This procedure is a direct application of the permutation importance measure defined by Breiman [41]. In the fourth step, the features are ordered in decreasing order according to this score, and the first 50 of them are selected. In the fifth step, the two operating inputs are added, and the set is completed to 52 inputs.

This design has three important details. The first one is that the stage where the ranking is produced and the stage where the subset is used are separate from each other. The ranking is computed once in the third stage and is kept fixed in the whole grid of the fourth stage, so a new selection is not made for each model, and the comparison between the models stays fair. The second one is that the selection is based on permutation instead of tree-based impurity decrease. The impurity decrease is biased towards features with high cardinality, while the permutation importance does not carry this bias because it measures the performance loss directly. The third one is that the selection has a redundancy-reducing side effect. Because the amplitude-based features of the same sensor are strongly related to each other, when one of them is shuffled, the model compensates the information from its twin and the measured drop stays small. For this reason, the upper part of the ranking is mostly filled with features that carry information independent from each other, and the set of 52 inputs keeps almost all the discriminative information carried by the set of 299 inputs. The content of the TOPK_XAI set and the results obtained with this set are discussed in detail in Section 3.4 and Section 3.6.

### 2.7. Performance Metrics

Because of the class imbalance, the primary metric has been set as *macro-F1*. The *Accuracy* is reported as the secondary metric. In the definitions given between Equations (1) and (5), *TP* indicates the number of true positives, *TN* the number of true negatives, FP the number of false positives, and *FN* the number of false negatives, *C* indicates the number of classes, *N* the number of samples, *y* the true label.

*Accuracy* = (*TP* + *TN*)/(*TP* + *TN* + *FP* + *FN*) (1)

*Precision* = *TP*/(*TP* + *FP*) (2)

*Recall* = *TP*/(*TP* + *FN*) (3)

*F1* = *2* × *Precision* × *Recall*/(*Precision* + *Recall*) (4)

*macro-F1* = (*1*/*C*) × *Σ F1*(*c*) (5)

In addition to these metrics, the *Precision*, *Recall* and *F1* values per class, the one-versus-rest area under the curve values, the multiclass logarithmic loss and the normalized confusion matrix are reported. In order to measure the dependence of the results on the random split, the grouped split has been repeated with five different seed values, and the mean and the standard deviation have been given. The significance of the difference between the best representatives of the ensemble and deep branches has been tested with a paired McNemar test on the same test set.

### 2.8. Explainability Analyses

Four separate analyses have been carried out in order to quantify which information the models rely on. The first one is the single feature permutation importance. In this measure, which has been defined by Breiman in the random forest context, each feature is shuffled in turn on a subsample taken from the test set, and the mean drop in the accuracy is recorded [41]. The second one is the group permutation. The columns that belong to the same sensor or to the same domain are shuffled together, and the contribution is measured at the sensor and domain levels. This extension removes the problem that the contributions of strongly related features look smaller than they are when they are shuffled one by one. The third one is the SHAP analysis applied to the best tree-based model. This framework, which has been based on the Shapley values of game theory by Lundberg and Lee, decomposes the feature contributions in an additive and consistent way [42]. The mean absolute SHAP value per feature is computed by using the exact and fast solution developed for tree ensembles [43]. The fourth is the integrated gradient method applied to the convolutional networks. In this method, which satisfies the sensitivity and implementation invariance axioms of Sundararajan et al., the gradients computed at 64 intermediate steps from the zero baseline towards the input are integrated, and the contribution of each input channel and of each time–frequency cell to the decision is extracted [44]. In addition, the separability of the scalar feature space has been visualized with the t-SNE embedding proposed by van der Maaten and Hinton [45], and the correlation structure of the most important features has been examined.

### 2.9. Ablation and Robustness Experiments

Five separate experiments have been carried out in the fifth stage. The operating input ablation measures how much of the performance comes from the vibration and acoustic signals and how much of it comes from the throttle level and the motor class. The sensor ablation compares the configurations of microphone only, accelerometer only, gyroscope only, inertial measurement unit and all sensors. In addition, an inertial axis subset ablation and a frequency representation ablation with cepstral and envelope spectrum features have been carried out with the same reference classifier, and a stationarity audit of the windows is reported in Section 3.1. The cross-motor experiment trains on one motor class and tests on the other one, which tests whether the model learns the physics of the fault or the traces specific to the motor. The repeated split experiment and the McNemar test provide the statistical verification. The reason why the McNemar test has been chosen is that, in the comparative review made by Dietterich, the probability of the type-one error of this test has been found low for algorithms that can be trained only once because of their high training cost [46]. In the same review, it has been shown that the test for the difference of two proportions and the paired *t* test based on random training and test splits produce a high type-one error. Finally, the inference latency and the disk size of each model have been measured, and the deployment cost has been reported. In the ablation experiments, a fixed reference classifier has been used so that the comparison between the variants can be fair. This reference model is an XGBoost classifier with 400 trees and a depth of 8, and it is trained without applying the prior correction. For this reason, the absolute values in the ablation tables differ slightly from the best model values in the main comparison table, but the relative ordering between the variants is preserved.

## 3. Results

### 3.1. Dataset and Signal Characterization

**Figure 3:**
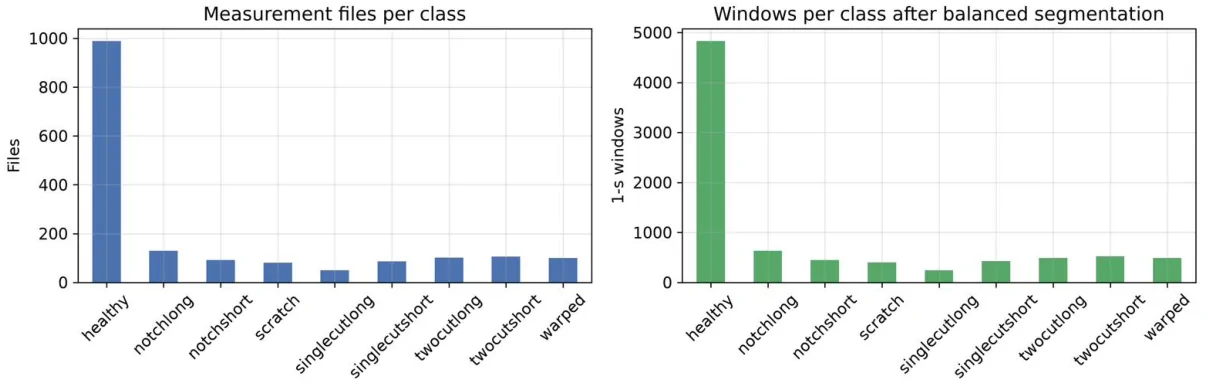
Measurement files and 1 s windows per class after balanced segmentation.

Figure 3 shows the number of measurement files per class and the number of windows obtained after the balanced segmentation side by side. The fact that the distribution in the left panel and the distribution in the right panel have the same shape reveals that the class-adapted step size keeps the ratio between the number of files and the number of windows constant. The healthy class is the most crowded group with 4831 windows, and it is about twenty times larger than the single long-cut class with 241 windows. This ratio shows that the accuracy metric alone would be misleading and that choosing macro-F1 as the primary metric is necessary.

**Figure 4:**
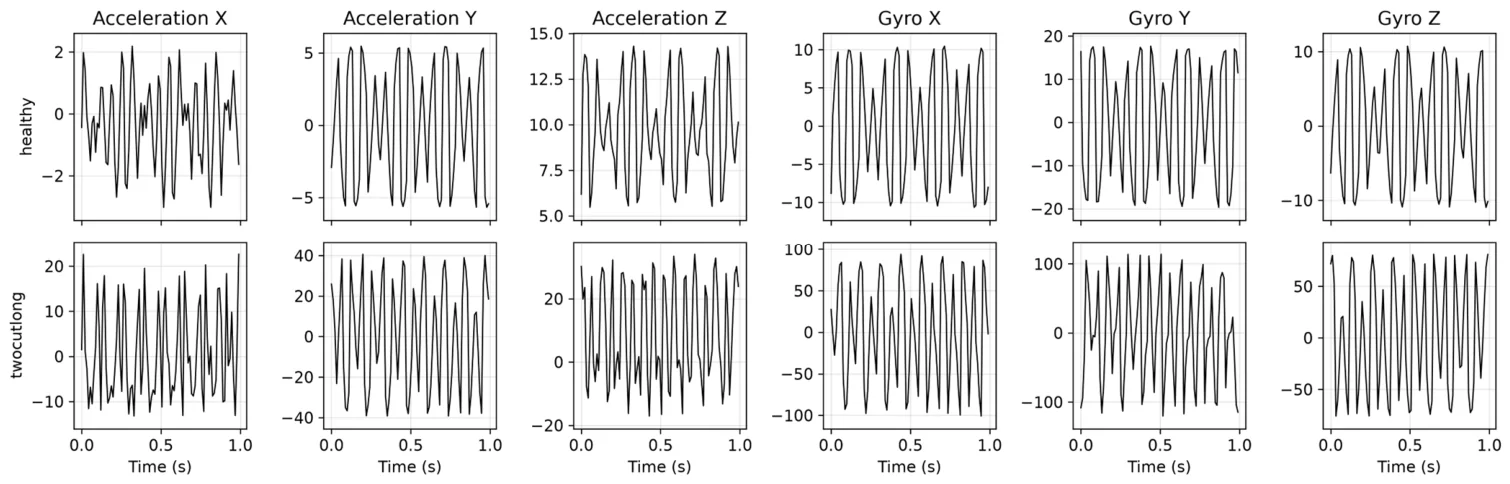
Representative raw 1 s windows of the six inertial channels for the healthy and the two long cut conditions at the same throttle level and motor class.

Figure 4 gives representative raw 1 s windows of the six inertial channels for the healthy and the two long-cut conditions recorded at the same throttle level and with the same motor class. Each panel has its own vertical scale. The windows are taken from the files 0467_healthy_980_100 and 0595_twocutlong_980_100, which have the median vibration level of their class at this operating point. The figure allows the amplitude levels of the two conditions to be compared before any transform is applied, and it shows the form of the signal that the models receive at the sampling rate of 90.9 Hz. The vertical axis of each panel is scaled separately, so the amplitude ranges of the two conditions have to be read from the axis ticks. Accordingly, both conditions show a periodic oscillation with about 10 cycles per second on all six channels, and the amplitude of this oscillation stays approximately constant inside the window, which is consistent with the constant throttle command inside a file and supports the assumption of approximate stationarity inside a 1 s window made in Section 2.4. The difference between the two conditions appears mainly as a change of the amplitude level. The peak-to-peak range of the two-cut long condition is about 10 times that of the healthy condition on the Gyro X and Gyro Y channels and about six times on the Acceleration Y channel, while the mean of the Acceleration Z channel stays near the gravity level in both conditions. Because the shaft frequency is above the Nyquist frequency at this throttle level, the oscillation seen in the figure is the aliased and low-frequency modulation component of the rotating forcing and not the shaft order itself, which is expected at this sampling rate.

In order to check the assumption of approximate stationarity inside a 1 s window, four tests have been applied to 1964 windows taken from 400 randomly selected measurement files. Each window has been divided into two halves, and the equality of the variances of the halves has been tested with an F test, and the equality of their means with a Welch *t* test. In addition, the absence of a trend has been tested with the reverse arrangements test, and the level stationarity has been tested with the KPSS test. Table 5 gives, for each channel, the share of the windows for which the test does not reject stationarity at the 5 percent level. Accordingly, the mean is stable in 94.7 to 97.2 percent of the windows of the inertial channels, and the KPSS test does not reject level stationarity in 93.0 to 95.9 percent of them, while the variance is stable in 83.0 to 86.8 percent. The microphone channel gives the lowest values, with 0.680 for the trend test and 0.865 for the mean test. It is thought that the lower values of the microphone come from the automatic gain control of the module, which changes the gain inside a window. These results show that the assumption of wide sense stationarity inside a window holds for the large majority of the inertial windows, and that the remaining windows do not carry a systematic trend that could be confused with a fault signature. The tests do not exclude cyclostationary behavior, because a periodic modulation of the variance is not detected by a two half comparison, and this point is discussed in Section 3.8.

**Figure 5:**
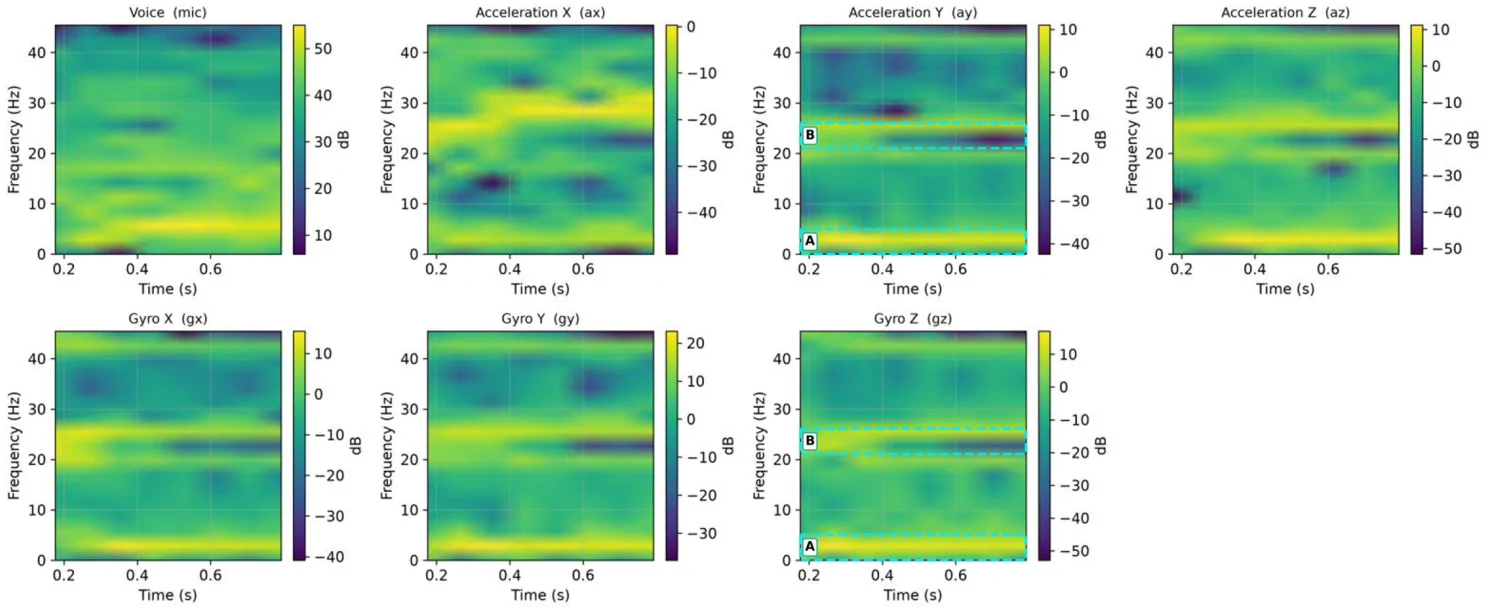
STFT spectrograms of all seven signals for the healthy condition. A marks the 0–5 Hz dominant band, and B marks the 22–25 Hz low-energy notch.

**Figure 6:**
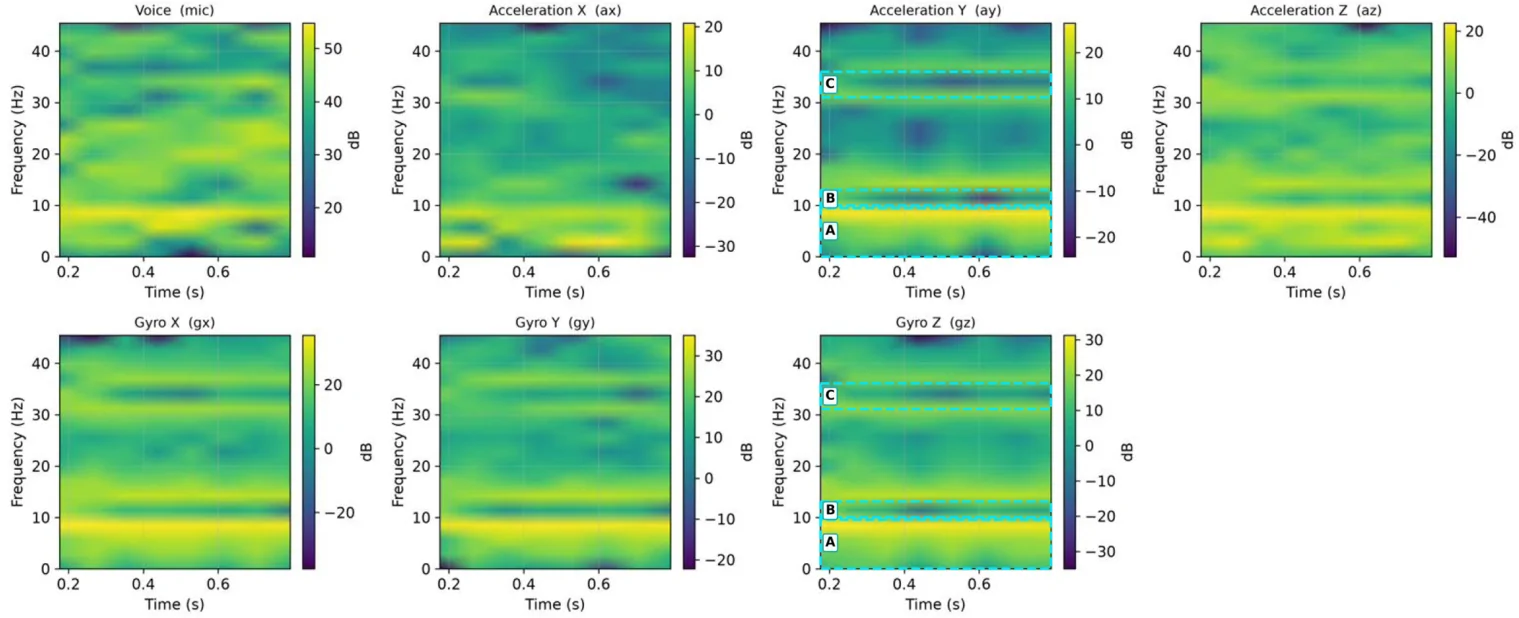
STFT spectrograms of all seven signals for the two cut long condition.

Figure 5 and Figure 6 give the short-time Fourier spectrograms of all seven signals for the healthy and the two long cut conditions. In both figures, the regions marked with dashed rectangles on the Acceleration Y and Gyro Z channels show the places where the difference between the two conditions can be followed with the naked eye. In the healthy condition, the dominant energy band marked with A is compressed between about 0 and 5 Hz, and a clear low-energy notch appears in the range of 22 to 25 Hz, marked with B. This notch is caused by the fact that the periodic forcing produced by a balanced propeller leaves very few components in this band.

In the two long cut condition, the same regions move in a clear way. The dominant band marked with A widens to the range of about 0 to 10 Hz, the low-energy notch marked with B moves down from around 22 Hz to the range of 10 to 12 Hz, and a secondary band marked with C appears around 33 Hz. The mass asymmetry created by the two separate cuts on the blade shifts the forcing per revolution towards lower frequencies and enriches the harmonic structure. The same shift can also be followed on the Gyro X and Gyro Y channels. It should be noted that these bands are observed at a sampling rate of 90.9 Hz, so they cannot be assigned to particular shaft orders, and they reflect the aliased and low-frequency modulation content of the rotating forcing as explained in Section 2.4. On the other hand, the difference between the two conditions is much weaker on the microphone panels. This visual observation shows that the damage leaves a clearer trace on the inertial channels than on the acoustic channel, and it gives a picture that is consistent with the sensor ablation and explainability results in the following sections.

**Figure 7:**
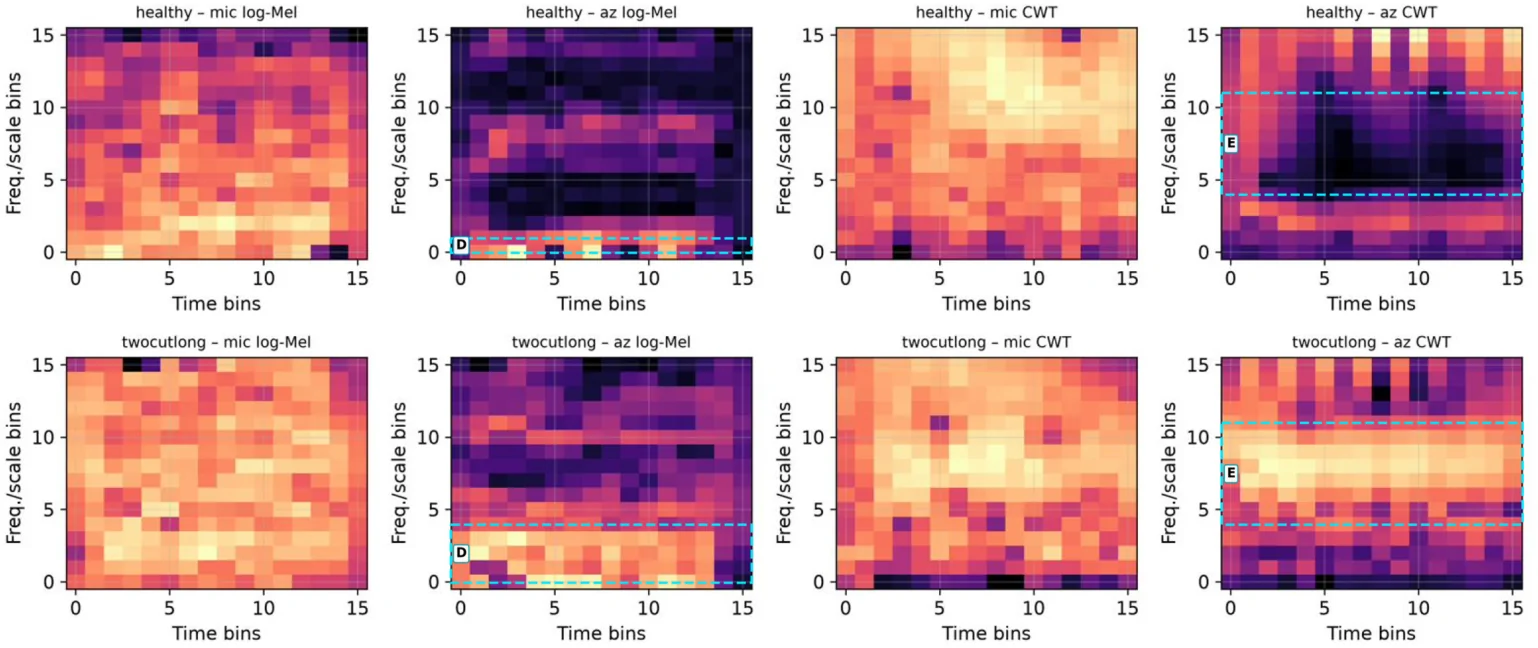
Examples of the raw time–frequency maps fed to the CNN branch. D marks the low-frequency band of the Acceleration Z log-Mel map, and E marks the mid-scale region of the Acceleration Z wavelet map. In all panels the magma colour scale is used, in which dark cells correspond to low and bright cells to high normalized magnitude, and each panel is scaled to its own range.

Figure 7 presents samples of the raw time–frequency inputs given to the convolutional networks. The upper row belongs to the healthy condition, and the lower row belongs to the two long cut condition. The two marked regions in the figure show how the observation based on the spectrograms is carried into the tensor representation. In the logarithmic spectrogram map of the Acceleration Z channel, the bright band marked with D covers only the zeroth- and first-frequency bins in the healthy condition, while it widens up to the fourth bin in the two long cut condition. This is the counterpart in the tensor space of the dominant band that spreads between 0 and 10 Hz in the previous figures. In the wavelet scalogram of the same channel, the region marked with E between the fourth and the eleventh scale bin is a dark low-energy area in the healthy condition and turns into a bright high-energy area in the two long cut condition. This inversion is the strongest visual difference between the two conditions. On the other hand, the microphone panels do not present a structure that can be separated easily between the two conditions in either the logarithmic spectrogram or the wavelet representation. The fact that the maps have been reduced to a size of 16 by 16 lowers the computational load, but as will be seen in the following sections, it also limits the capacity of the convolutional networks to learn this discriminative pattern.

### 3.2. Model Comparison

Table 6 gives the test set performance of 10 models sorted according to the macro-F1 metric. All seven methods in the ensemble branch have passed all three architectures in the deep branch. The highest macro-F1 value of 0.7937 belongs to the extra trees model, and this model has reached an accuracy of 0.8769 and a macro precision of 0.8868. The random forest takes the second place with a macro-F1 of 0.7838. The stacked generalization and LightGBM are competitive in terms of accuracy but stay behind in terms of macro-F1, which shows that these models make more errors on the minority classes. The best result in the deep branch belongs to the ResNetTF architecture with a macro-F1 of 0.4912, and this value is at about two-thirds of the weakest ensemble method.

It should be underlined that this comparison is limited to three compact architectures with 26,553 to 82,665 parameters that operate on maps reduced to 16 by 16 cells. Therefore, the result shows that the ensemble branch outperforms the tested convolutional configurations on this dataset, and it does not show that deep learning methods are inferior in general. Larger architectures, higher-resolution maps, pretrained backbones and one-dimensional networks applied to the raw signal have not been tested, and these are left to a separate study as stated in Section 4.

**Figure 8:**
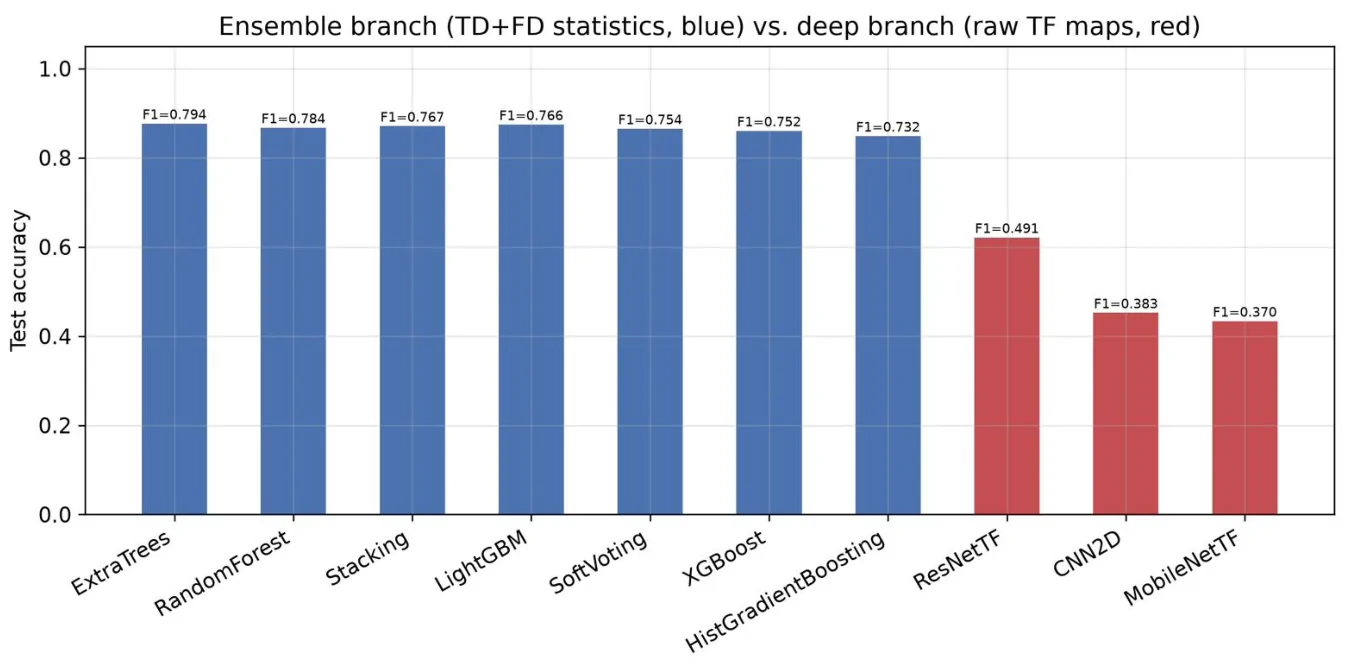
Test accuracy of the ensemble branch and the deep branch. Macro-F1 values are annotated above each bar.

Table 6 shows a clear separation in terms of the training times. The random forest is trained in 20.4 s, while the stacked generalization needs 906.8 s. The fact that the stacked generalization stays behind the random forest in terms of macro-F1 in return for 40 times more time shows that the high-level combiner does not provide an additional gain on this dataset. The training times of the deep networks are between 17 and 22 s, but these times come from the fast start of the early stopping and have to be evaluated together with the low performance. Figure 8 presents the same results visually. The blue bars represent the ensemble branch, and the red bars represent the deep branch. The gap between the two branches is about 0.25 accuracy points, and it is too large to be explained by a random fluctuation. The macro-F1 labels above the bars also show that the accuracy ordering and the macro-F1 ordering do not always overlap.

### 3.3. Per-Class Performance

Table 7 gives the precision, recall and F1 values of the best model per class. The healthy class is caught almost perfectly with a recall of 0.9938. The long-notch, short-notch and two short-cut classes show a strong performance with F1 values in the range of 0.84 to 0.90. Against this, two classes are clearly weak. In the single short-cut class, the precision is 0.9615, while the recall falls to 0.5556, which means that the model selects this class only when it is sure and misses about half of the samples. In the warped class, the precision is 1.0000, while the recall is 0.3400. Every prediction produced for this class is correct, but about two-thirds of the real warped samples are missed.

**Figure 9:**
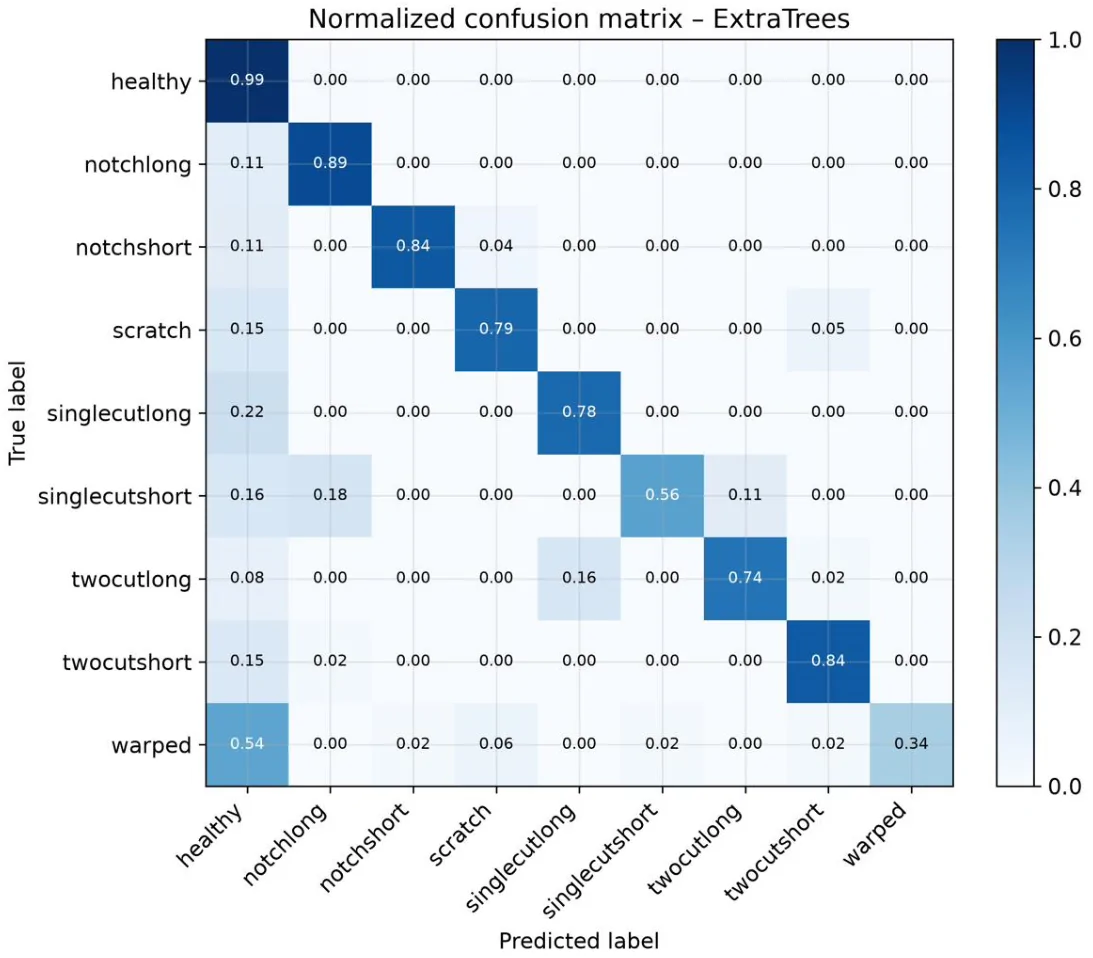
Normalized confusion matrix of the best model over the nine propeller conditions.

Figure 9 gives the normalized confusion matrix. The matrix shows that the errors of the weak classes are not distributed randomly. Fifty-four percent of the warped samples are classified as healthy. This result is physically meaningful, because warping is a deformation that does not create a material loss on the blade and that disturbs the mass distribution relatively little. Therefore, the vibration signature it produces stays close to that of the healthy propeller. In a similar way, 18 percent of the single short-cut samples are labelled as long notch and 16 percent of them are labelled as healthy. The fact that 16 percent of the two long-cut samples are classified as single long-cut suggests that the length of the damage and not the number of the damages is the dominant discriminative factor. The general tendency in the first column of the matrix reveals that the model shifts towards the healthy class in uncertain cases, and this is an unwanted bias in terms of safety.

**Figure 10:**
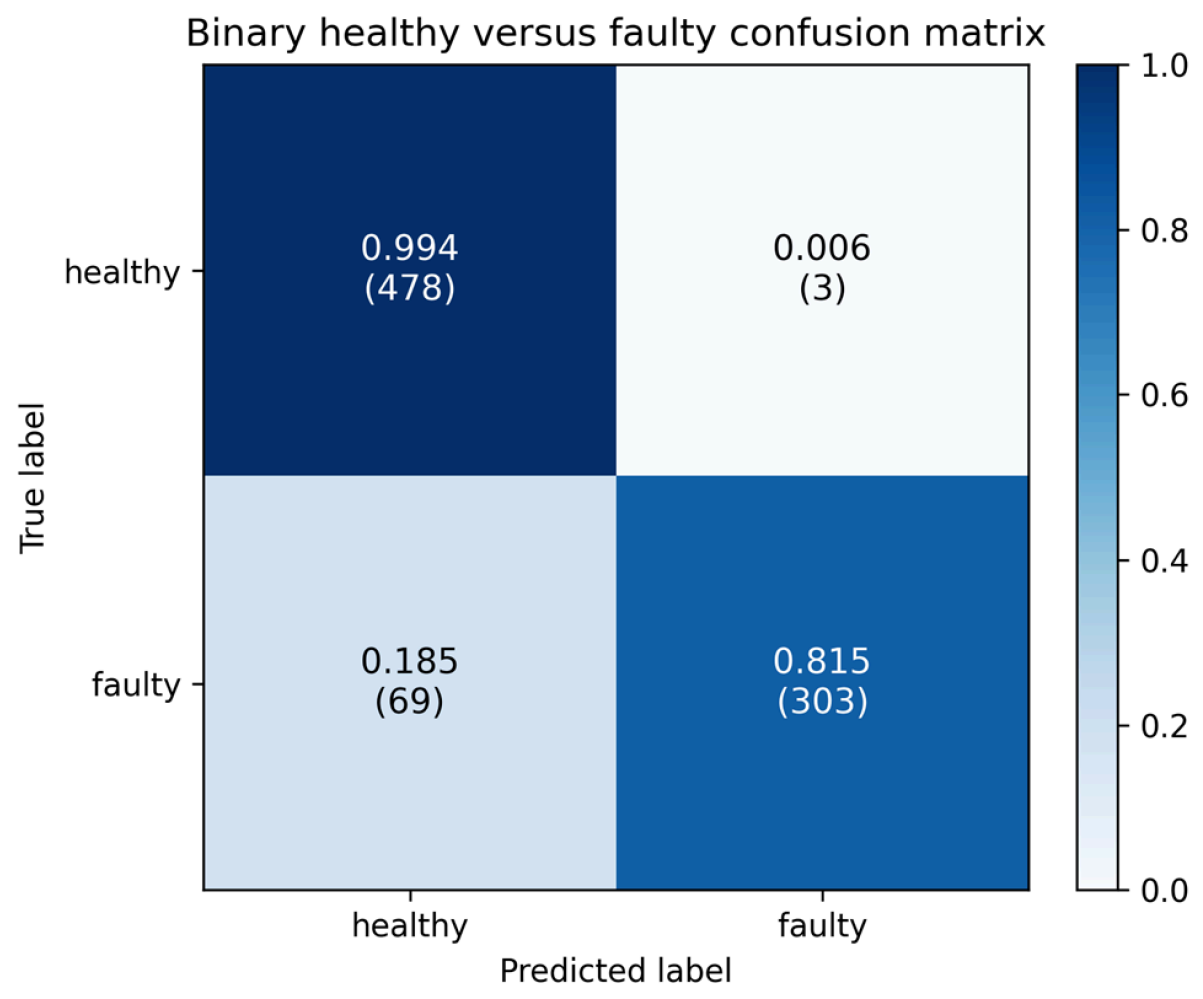
Binary healthy versus faulty confusion matrix obtained by collapsing the nine class predictions of the same model.

In order to place the difficulty of the nine-class problem in context, the predictions of the same model have been reduced to two classes as healthy and faulty, and the binary confusion matrix has also been computed. The reduction has been made by collecting all the labels other than healthy in the nine class predictions into a single faulty class, so no new model has been trained, and the same test partition has been used. Figure 10 gives the result. In the binary setting, the accuracy reaches 0.9156, and the macro-F1 reaches 0.9119. For the faulty class, the precision is 0.9902, and the recall is 0.8145, while for the healthy class, the precision is 0.8739, and the recall is 0.9938. Only three of the 481 healthy windows in the test set are marked as faulty, which means that the false alarm rate stays below the level of 1 percent. Against this, 69 of the 372 faulty windows are classified as healthy, and the missed fault rate stays at the level of 18.5 percent.

This comparison reveals two points in a clear way. The first one is that determining the presence of a fault is a relatively easy task, and the proposed approach reaches 91.6 percent accuracy on this task. The high values reported by many studies in the literature also belong to this binary setting [13,14,16,18]. The second one is that determining the type of the fault is a clearly harder task, and the accuracy falls to 0.8769 for the same model. This study targets the second and more detailed task, because in order to make a maintenance decision, not only the presence of the fault but also its type and its severity are needed. The high performance obtained in the binary setting shows that the values observed in the nine-class setting come from the nature of the problem and not from a weakness of the model.

**Figure 11:**
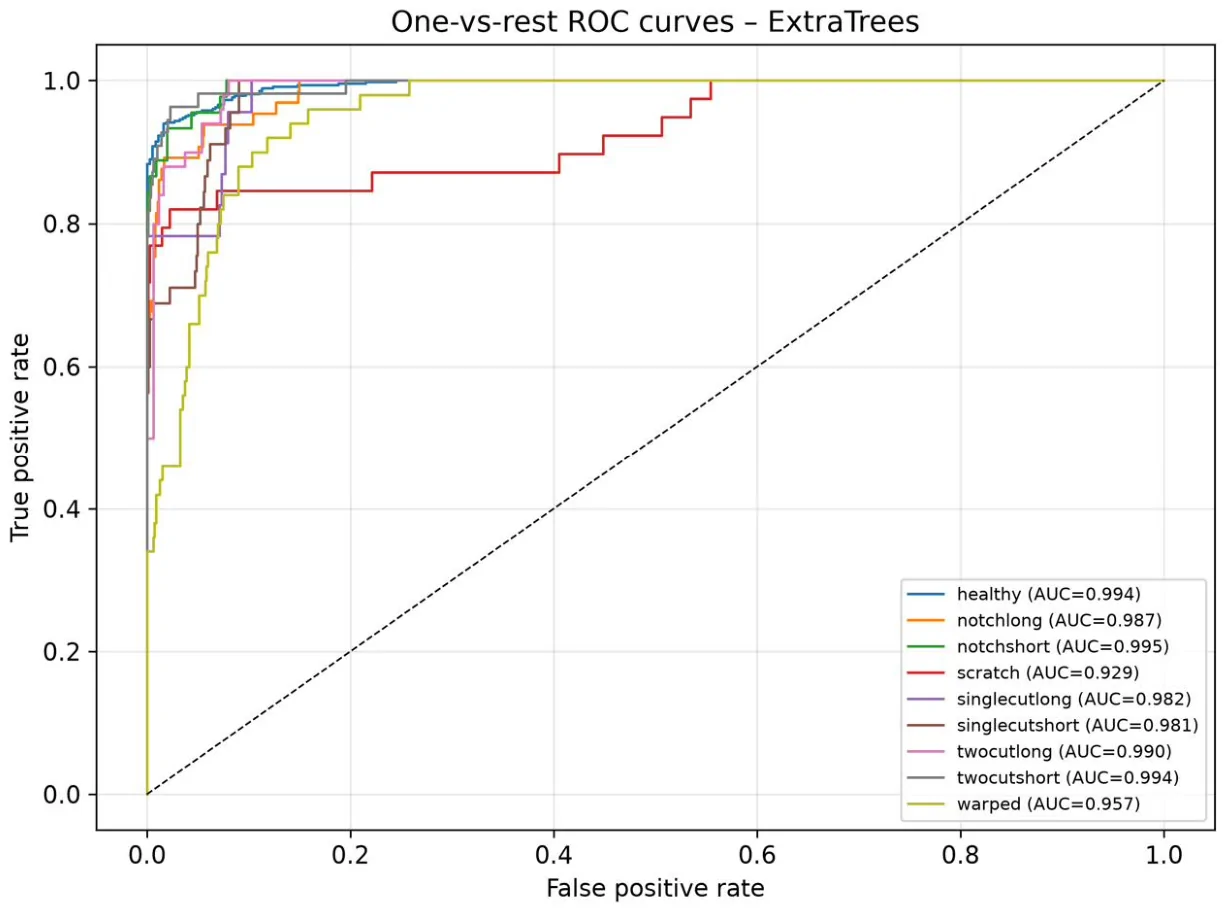
One-versus-rest ROC curves of the best model.

Figure 11 gives the one-versus-rest receiver operating characteristic curves. The area under the curve values are high in all classes, and the overall macro value is at the level of 0.9787. This apparent contradiction between the weak recall in the confusion matrix and the high value of the area under the curve shows that the problem does not come from the inability of the model to separate the classes but from the fact that the decision threshold is at a point that favors the healthy class. In practice, tuning the threshold again according to the cost of a missed fault can clearly increase the recall in the warped and single short-cut classes.

### 3.4. Results of Feature Set and Model Grid

Table 8 gives all 33 feature and model pairs. The highest macro-F1 value has been obtained with the combination of the XAI-ranked subset of 52 inputs and the stacked generalization, and it corresponds to a macro-F1 of 0.8027 and an accuracy of 0.8816. The highest accuracy has been observed as 0.8839 in the combination of the time domain set with 155 inputs and the extra trees. The difference between the two pairs is negligible in statistical terms, but the first one uses only about one-third of the inputs. How the TOPK_XAI set is built has been explained step-by-step in Section Construction of the TOPK_XAI Feature Subset. When the content of this set is examined, it is seen that 40 of the 50 signal features belong to the time domain and 10 of them belong to the frequency domain. In terms of the channel distribution, the Gyro Z channel takes the first place in the list with 10 features, and it is followed by the acceleration vector magnitude, the microphone, the Gyro X, the Acceleration Y and the gyroscope vector magnitude channels with six features each. The fact that all nine channels are represented in the list shows that the selection is not concentrated on a single sensor and that the multisensor design is also preserved in the reduced set.

The most remarkable finding of the grid is that the time-domain set passes the frequency-domain set by a clear margin in every model. With the time-domain set, the macro-F1 values vary between 0.7587 and 0.8007, while with the frequency-domain set, they stay in the range of 0.6115 to 0.6788. Combining the two sets does not provide an improvement over the time-domain set. This shows that the Welch-based frequency-domain features used in this study are not discriminative enough in the narrow band obtained at a sampling rate of 90.9 Hz and that they behave like a noise source in the combined set. This finding is restricted to the specific frequency features of this study, and it does not mean that the frequency content of the vibration signal is uninformative in general. As explained in Section 2.4, the shaft orders are not resolved at this sampling rate, and a representation obtained with an angular reference may give a different ordering. The fact that the TOPK_XAI set gives an equal or better result with only 52 inputs compared with the 299 inputs of the TD + FD set also supports this interpretation.

**Figure 12:**
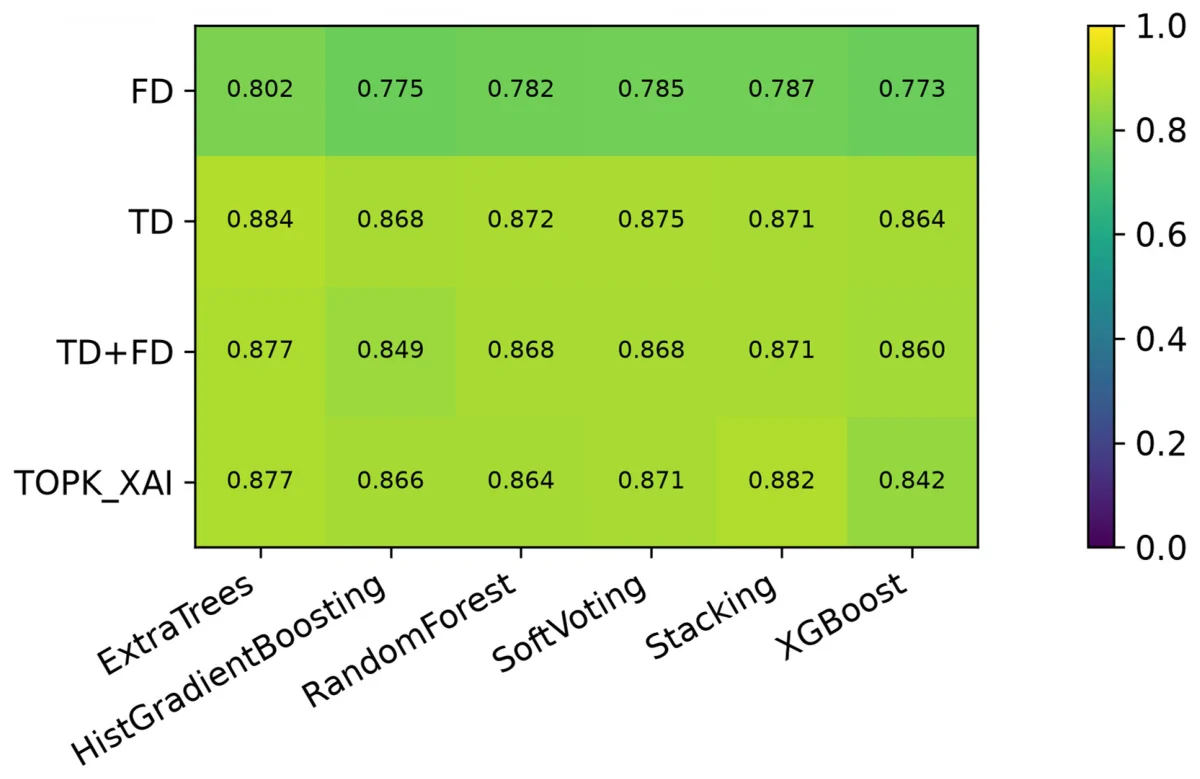
Test accuracy heat map of the ensemble branch.

**Figure 13:**
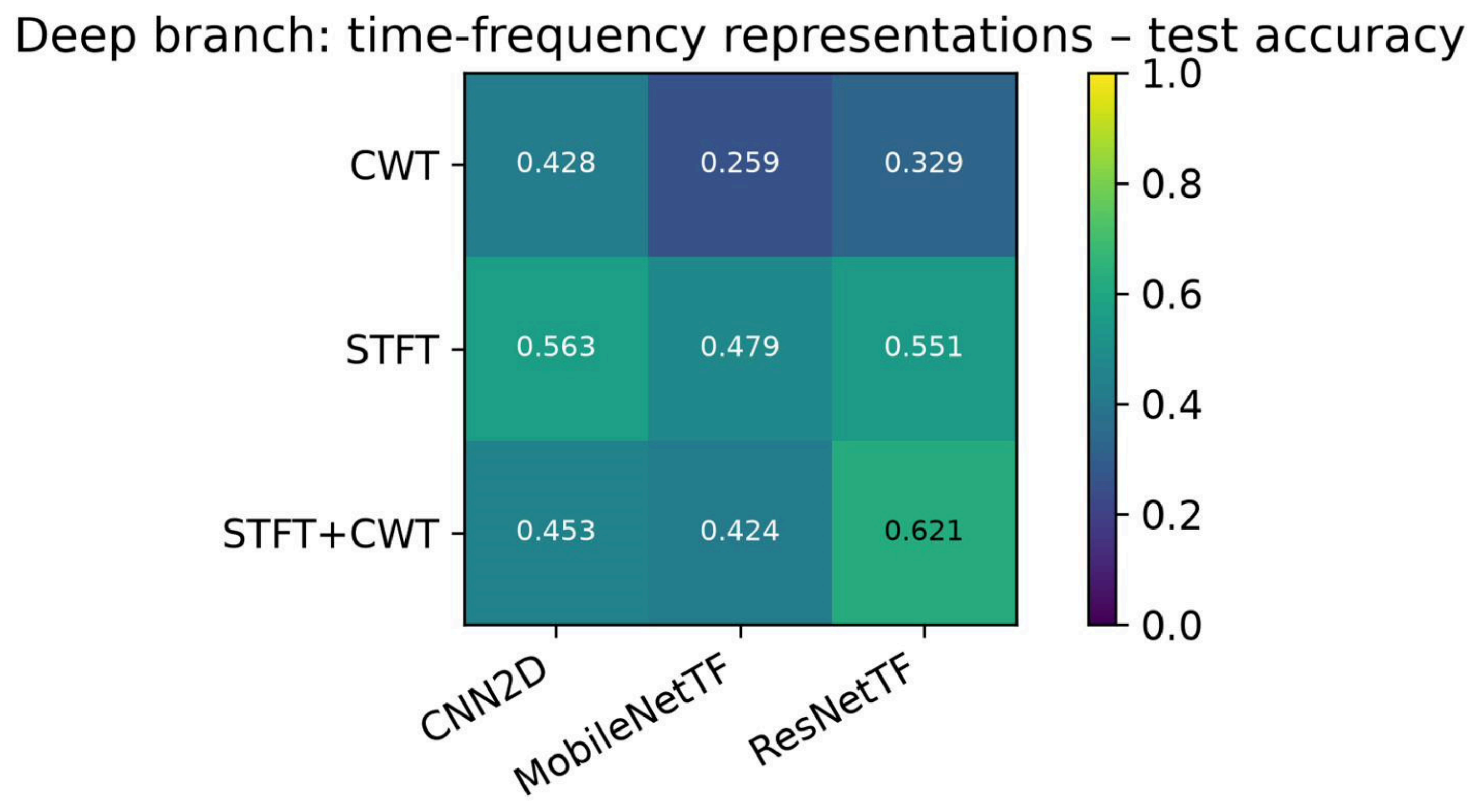
Test accuracy heat map of the deep branch.

**Figure 14:**
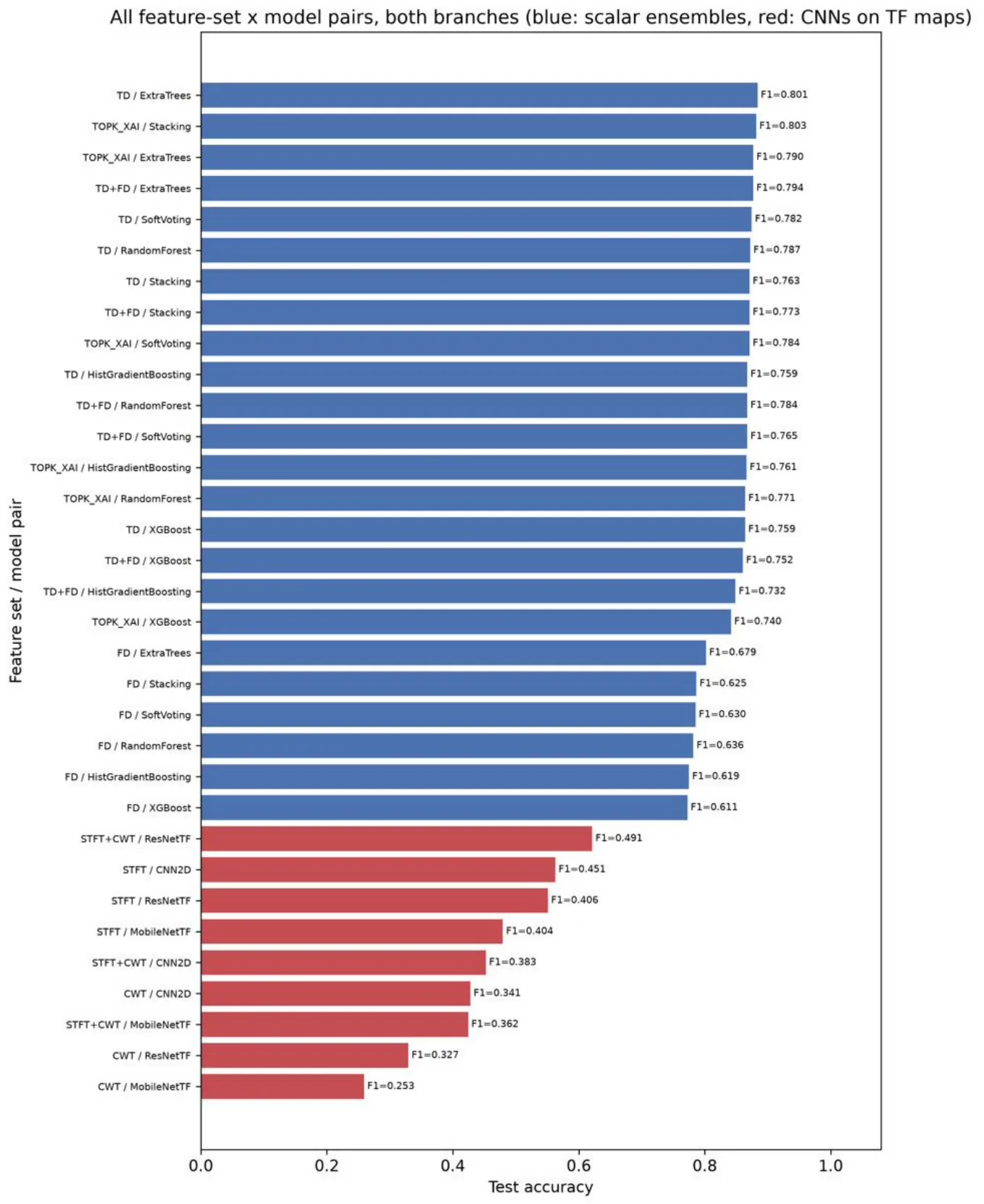
All feature set and model pairs across both branches.

**Figure 15:**
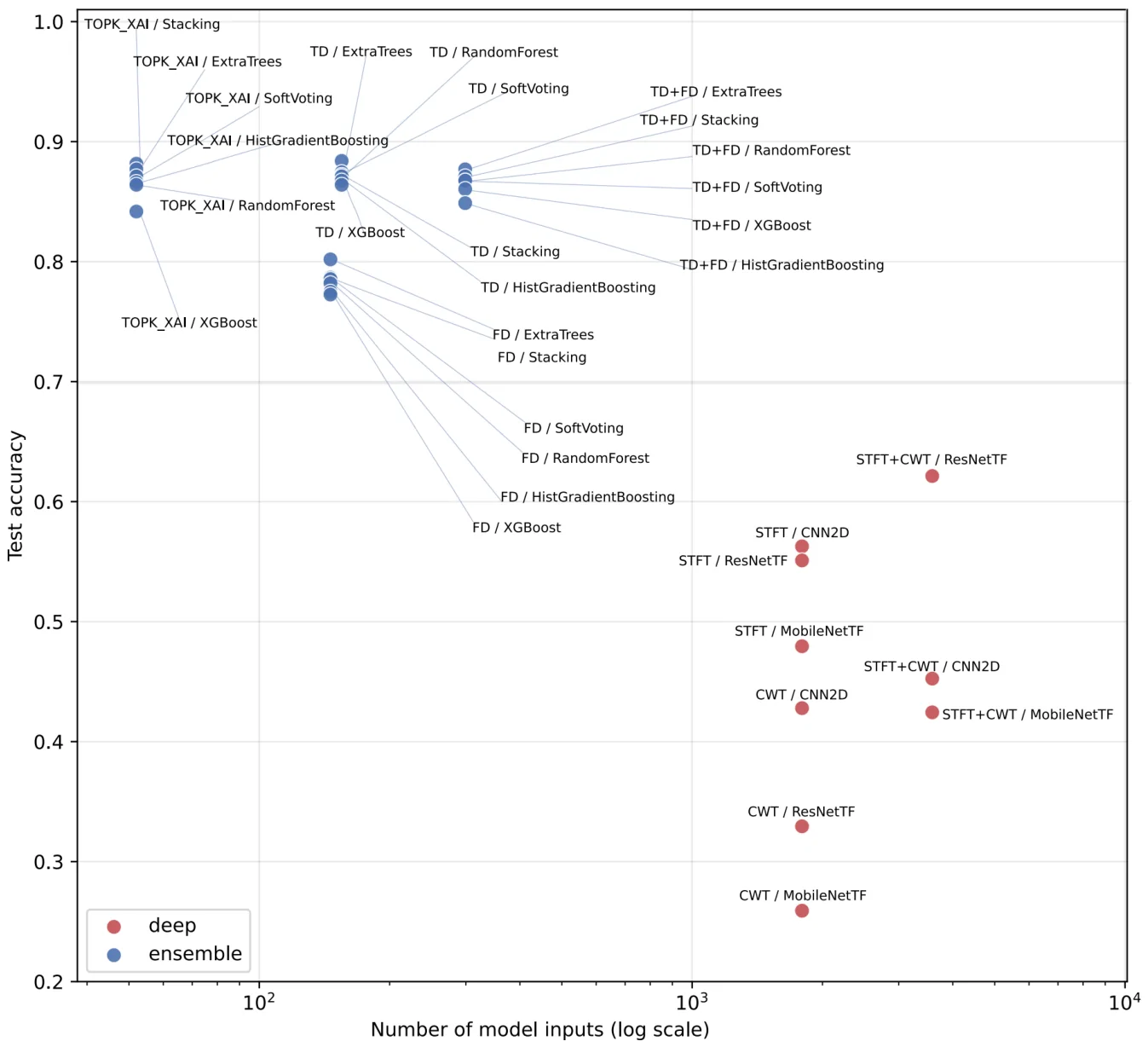
Test accuracy versus input dimensionality. Every feature set and model pair is labelled, and the labels are stacked in a single column on the right.

Figure 12 and Figure 13 give the heat maps of the two branches. In the map of the ensemble branch, the difference between the rows is clearly larger than the difference between the columns, which means that the choice of the feature set is more effective than the choice of the model. In the map of the deep branch, the highest value belongs to the ResNetTF pair where the STFT and CWT channels are used together. Using only the wavelet channels gives the worst result in all the architectures, and with MobileNetTF, the accuracy falls down to the level of 0.2591. In a problem with nine classes, this value is only twice the level of a random guess. Figure 14 combines all the pairs in a single sorted bar chart and reveals the separation between the two branches clearly. In the chart, all the ensemble pairs are placed above the best deep pair. Figure 15 plots the accuracy against the input dimension. In this chart, where the horizontal axis is logarithmic, the difference between the TOPK_XAI pairs with 52 inputs and the deep pairs with 3586 inputs is striking. Increasing the number of inputs by about 70 times does not raise the performance but lowers it. This result shows that learning a high-dimensional raw representation is difficult on a dataset with a limited number of samples.

### 3.5. Training Behavior of the Deep Networks

**Figure 16:**
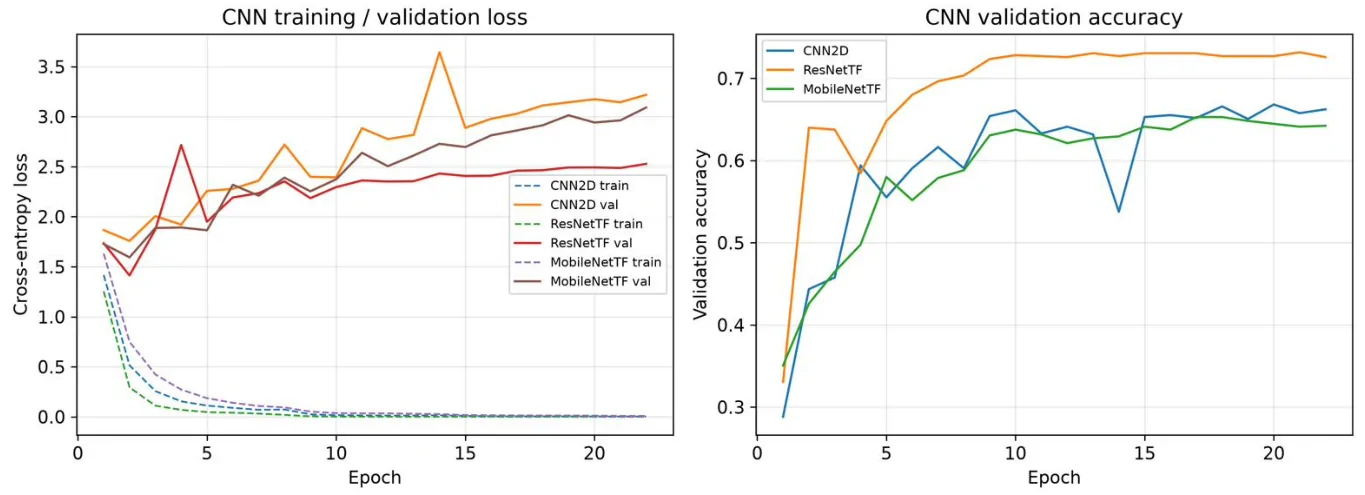
Training and validation curves of the three compact CNN architectures.

Figure 16 gives the training and validation loss curves and the validation accuracy curves of the three convolutional networks. In all three architectures, the training loss decreases regularly, while the validation loss reaches a plateau at an early epoch and then starts to rise. The opening between the training and validation curves shows that the networks learn details specific to the partition instead of a generalizable pattern on the maps of size 16 by 16. Early stopping with a patience of 20 epochs limits the overfitting, but the validation accuracy that is reached is lower than that of the ensemble methods. This behavior supports the interpretation that the high deep learning performance reported in our previous study [37] is largely attributable to the random split at the window level.

### 3.6. Explainability Results

**Figure 17:**
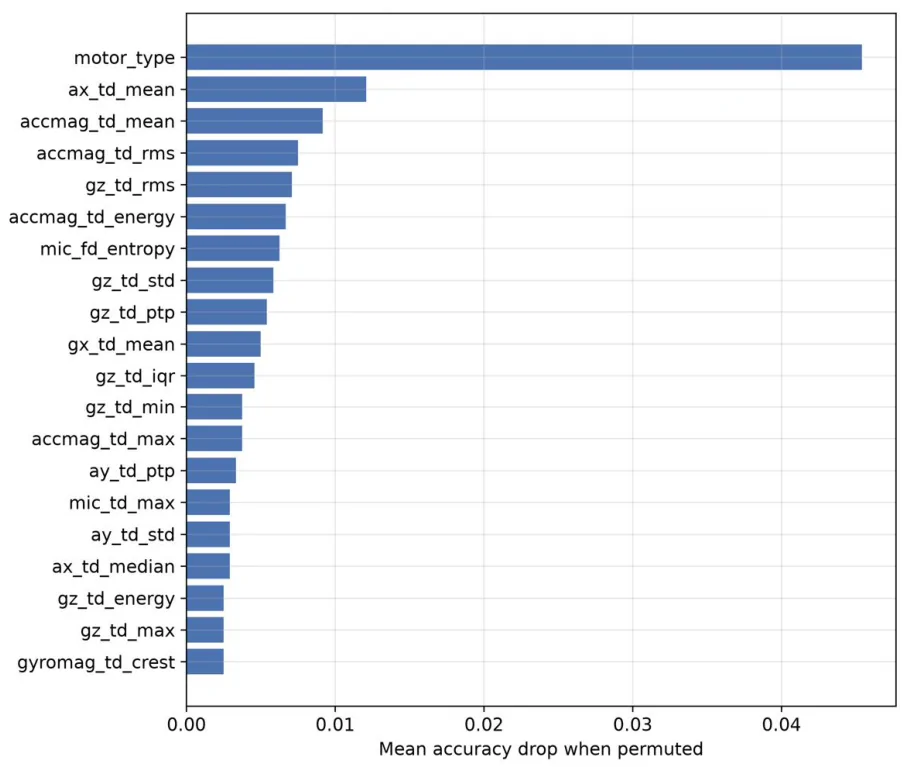
Top 20 features ranked by permutation importance.

Figure 17 gives the 20 features with the highest permutation importance. The motor class is at the top of the ranking, and it alone leads to an accuracy drop of 0.0454. It is followed by the mean of the Acceleration X axis with 0.0121, the mean of the acceleration vector magnitude with 0.0092 and the root mean square value of the acceleration vector magnitude with 0.0075. Only three of the first 20 features belong to the frequency domain. This distribution confirms, at the feature level, the superiority of the time domain seen in the grid results.

**Figure 18:**
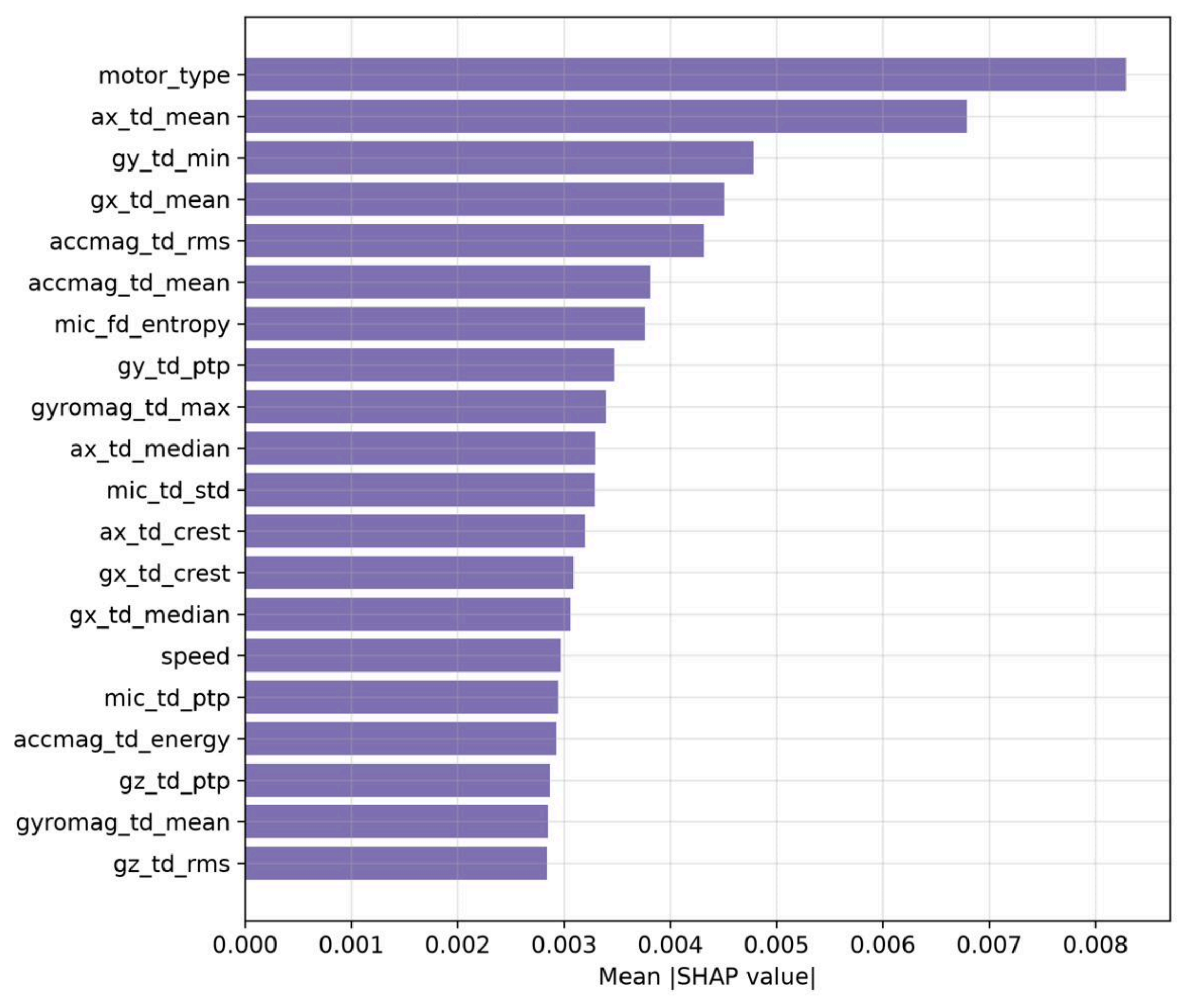
Top 20 features ranked by mean absolute SHAP value.

Figure 18 gives the SHAP values computed on the best tree-based model. The first places of the two methods overlap to a large extent. The motor class and the mean of the Acceleration X axis are in the first two places in both rankings. The SHAP ranking additionally brings forward the minimum and peak-to-peak values of the gyroscope axes. The agreement of the two methods shows that the obtained importance ranking is not a side effect of a single explanation technique.

**Figure 19:**
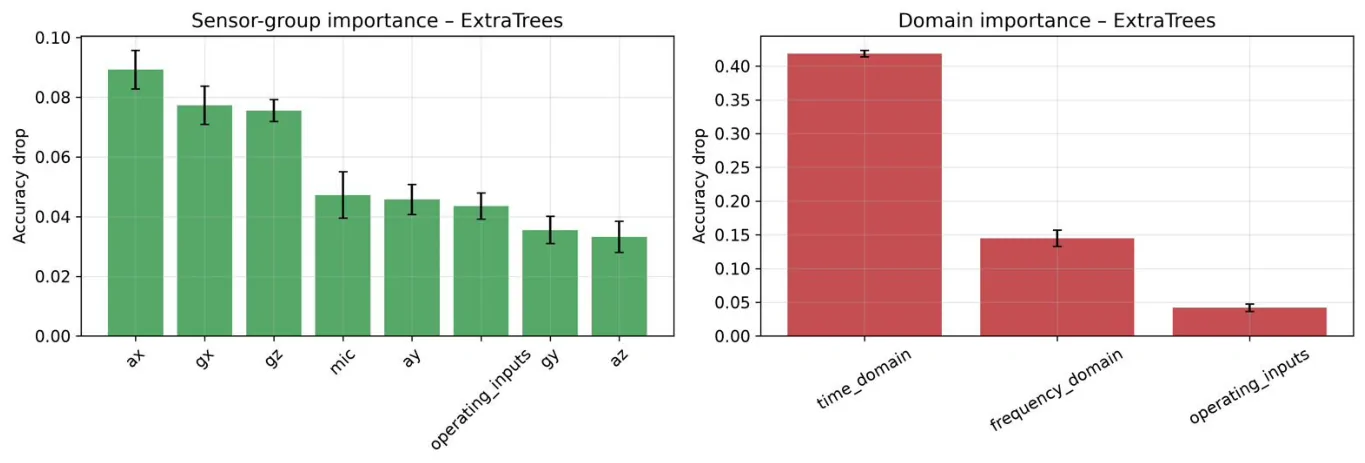
Group permutation importance at the sensor level and at the domain level.

Figure 19 gives the group permutation results at the sensor and domain levels, and Table 9 together with Table 10 gives the numerical values of the same results. At the sensor level, the highest contribution belongs to the Acceleration X axis with an accuracy drop of 0.0893. It is followed by the Gyro X axis with 0.0773 and the Gyro Z axis with 0.0755. The microphone is in the middle with 0.0473, and the Acceleration Z axis gives the lowest contribution with 0.0332. At the domain level, the separation is much sharper. Shuffling the time domain columns together drops the accuracy by 0.4185, while this value is 0.1448 for the frequency domain and 0.0420 for the operating inputs. The time domain statistics carry about three times more information than the Welch-based frequency-domain features used in this study.

**Figure 20:**
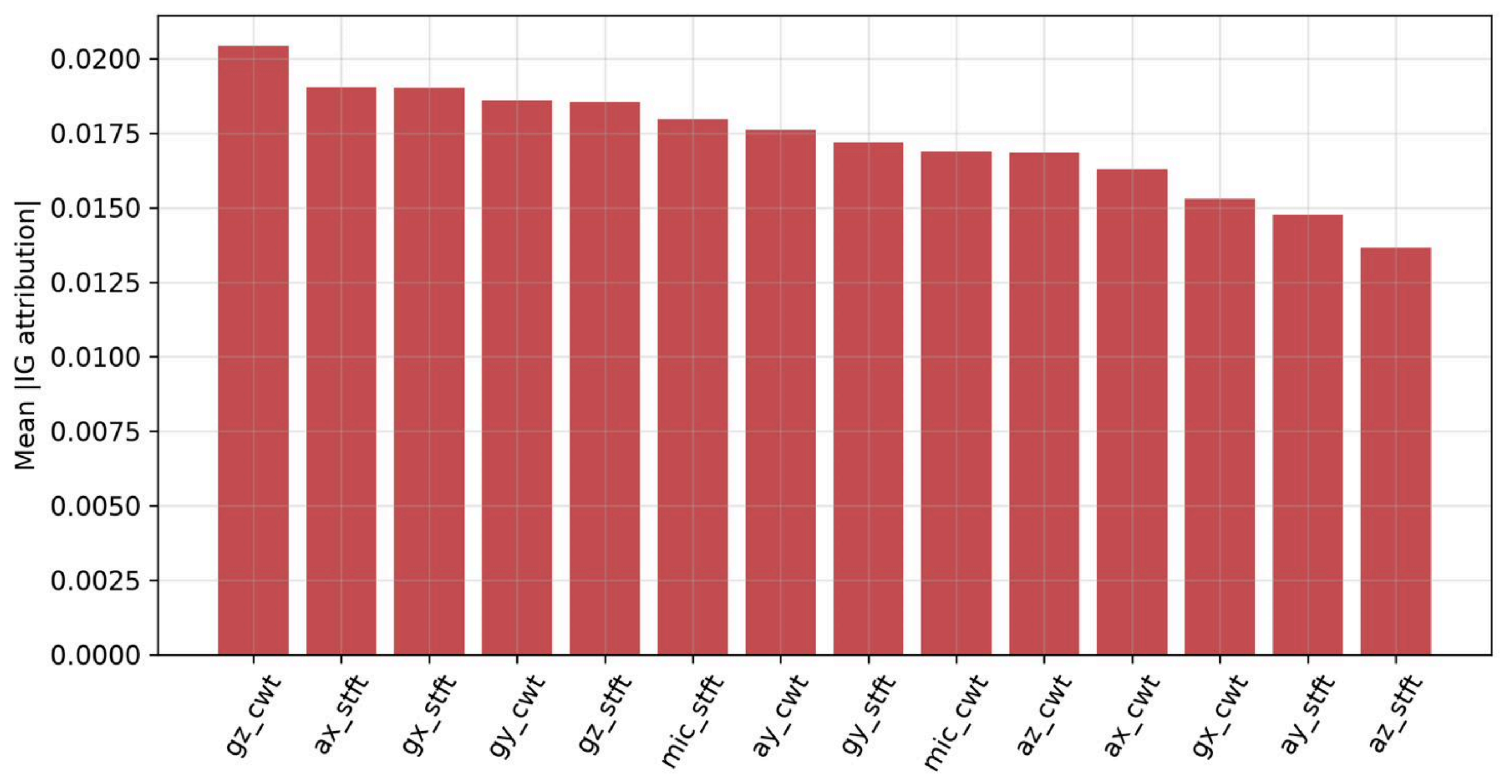
Integrated gradients channel importance for the best CNN.

Figure 20 gives the integrated gradient contributions of the input channels of the convolutional network, and Table 11 gives the same values numerically. The highest contribution of 0.0204 belongs to the wavelet channel of the Gyro Z axis, and the lowest contribution of 0.0137 belongs to the spectrogram channel of the Acceleration Z axis. The ratio between the highest and the lowest value is only about 1.5. This flatness shows that the network cannot focus on a particular channel and that it distributes the contribution almost equally over the 14 channels. Compared with the sensor importances of the ensemble branch, where the difference between the highest and the lowest is 2.7 times, it is understood that the convolutional networks cannot make a discriminative channel selection on this dataset.

**Figure 21:**
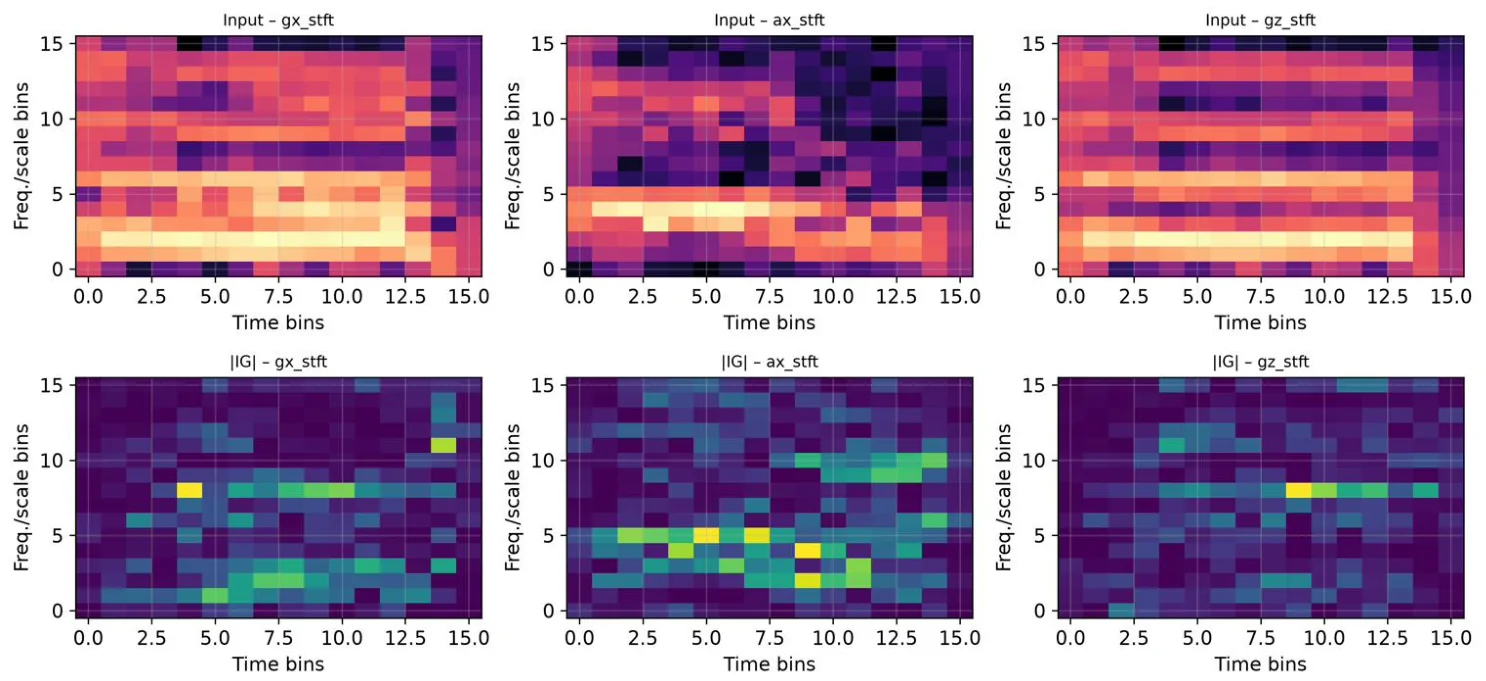
Integrated gradient attribution maps for the most strongly attributed test window.

Figure 21 gives the input map of three channels for the test sample with the highest total contribution together with the corresponding absolute contribution map. No concentrated and interpretable region appears in the contribution maps, and the contributions are spread over the time and frequency axes. This visual finding is consistent with the flatness at the channel level and suggests that the reduced maps of size 16 by 16 cannot preserve the discriminative local pattern.

**Figure 22:**
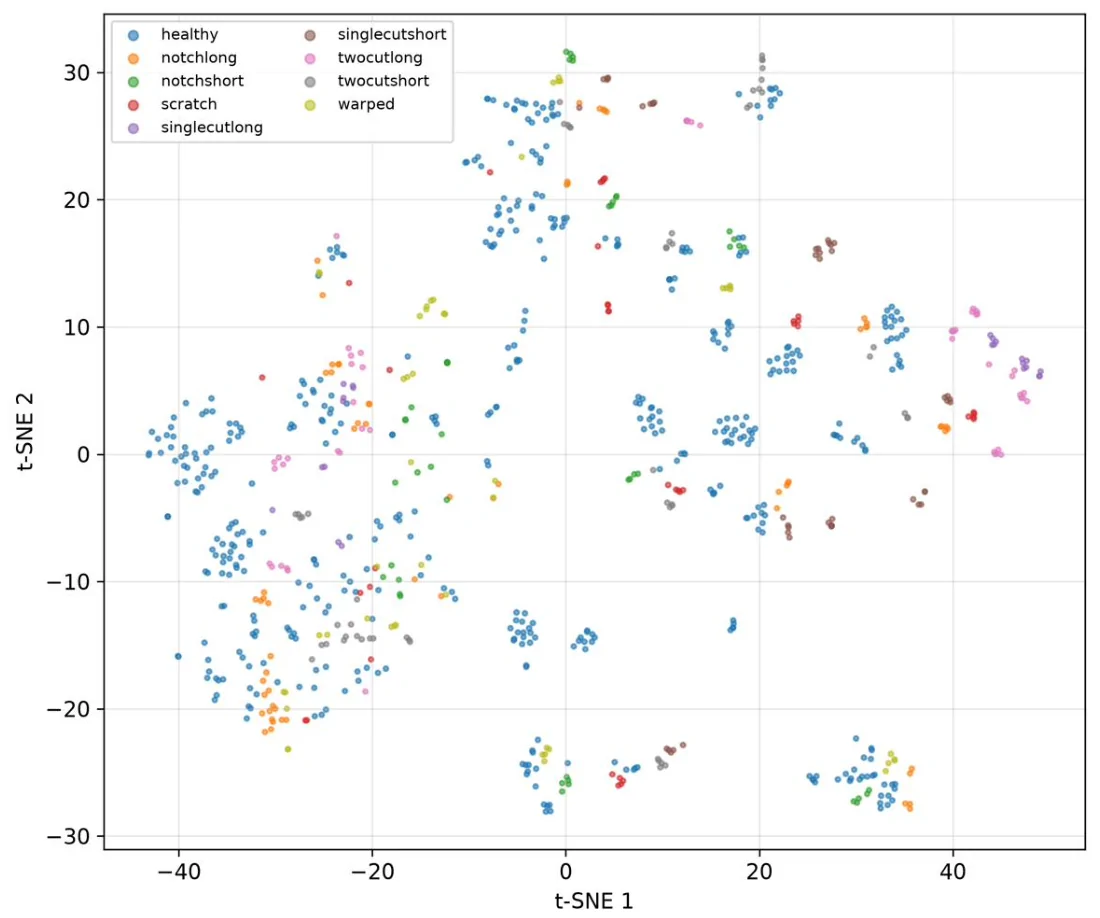
t-SNE embedding of the scalar feature space for the test windows.

Figure 22 gives the t-SNE embedding of the test windows in the scalar feature space. The healthy class forms a wide and scattered cloud, while the faulty classes appear as clusters embedded inside and at the edges of this cloud. The long-notch and short-notch classes form relatively separate clusters, while the warped class is largely mixed with the healthy cloud. This indicates that the confusion between warped and healthy in the confusion matrix is not a defect of the model but a natural result of the feature space.

**Figure 23:**
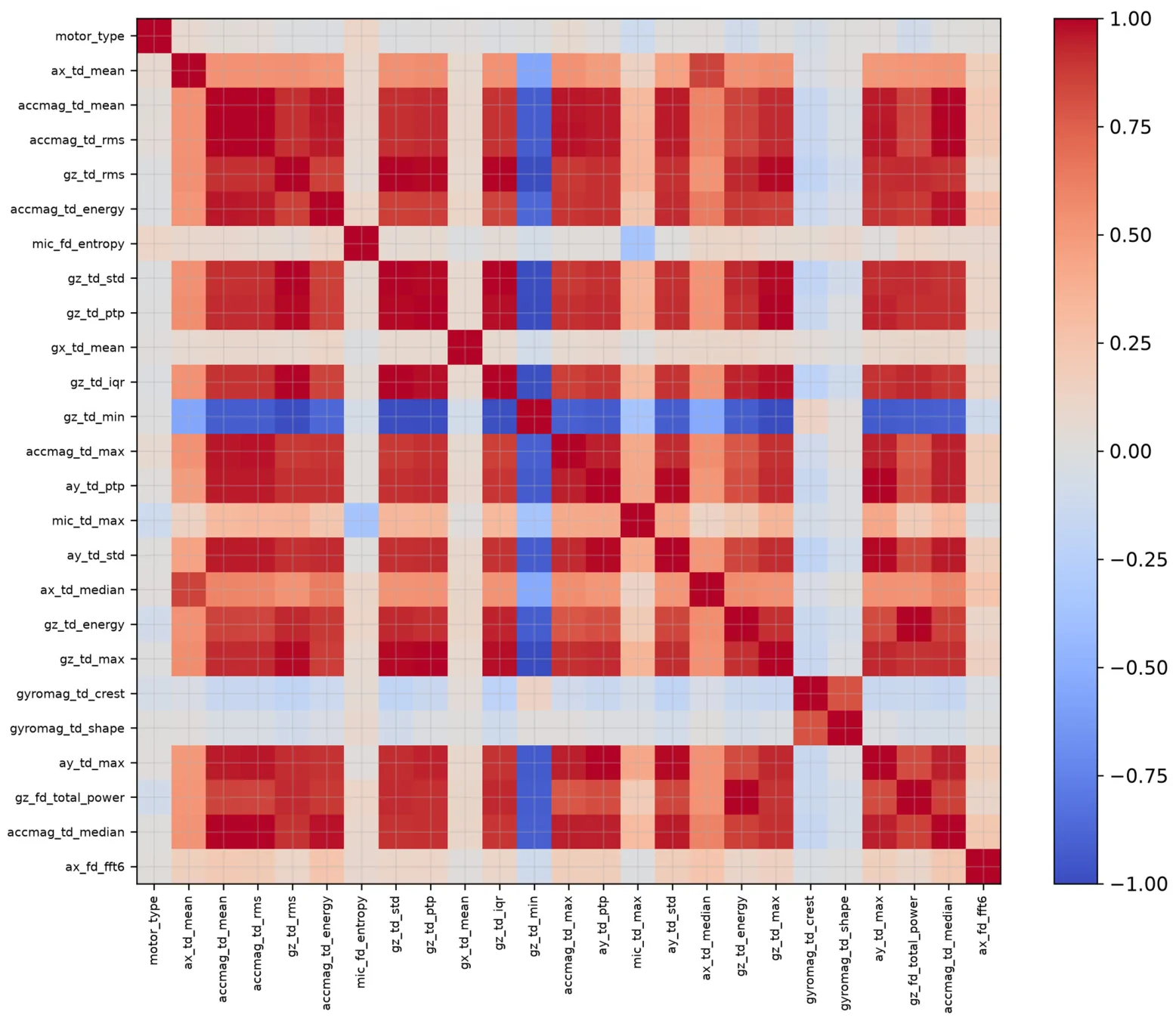
Correlation matrix of the 25 most important scalar features.

Figure 23 gives the correlation matrix of the 25 most important features. There is a strong positive correlation between the amplitude-based features that belong to the same sensor. For example, the mean, the root mean square and the energy of the acceleration vector magnitude are almost collinear. This structural redundancy explains why the TOPK_XAI set can reach the performance of the set with 299 inputs by using only 52 inputs.

When the four explainability analyses are read together, they support the feature–model matching principle of this study in a chain of four steps. First, the single feature permutation importance in Figure 17 and the SHAP values in Figure 18 produce the same top-ranked features with two different mechanisms, so the ranking used for building the TOPK_XAI subset is not an artefact of one technique. Second, the group permutation at the domain level in Table 10 shows that the time-domain statistics carry about three times the contribution of the Welch-based frequency-domain features, which explains why the time-domain sets take the first places in the grid of Table 8 and why the combined set does not improve over the time-domain set. Third, the correlation matrix in Figure 23 shows that the amplitude-based features of the same sensor are almost collinear, which explains why the 52-input subset reaches the performance of the 299-input set and why the tree ensembles, which are robust against correlated inputs, are the suitable learner family for this representation. Fourth, the integrated gradient analysis in Figure 20 and Figure 21 shows that the convolutional networks distribute their attribution almost uniformly over the 14 channels and over the time–frequency cells, and this flatness is the counterpart of the low performance of the deep branch. In other words, the tensor representation reduced to 16 by 16 cells does not present a local pattern that the convolutional filters can select, while the scalar representation presents a small number of dominant statistics that the tree ensembles can select. Accordingly, the matching of the scalar time-domain features with the tree ensembles is not only the empirically best pair of the grid, but also the pair whose decision behavior is explained consistently by all four analyses, and the reduced 52-input subset is the direct product of this chain.

### 3.7. Sensor Ablation

**Figure 24:**
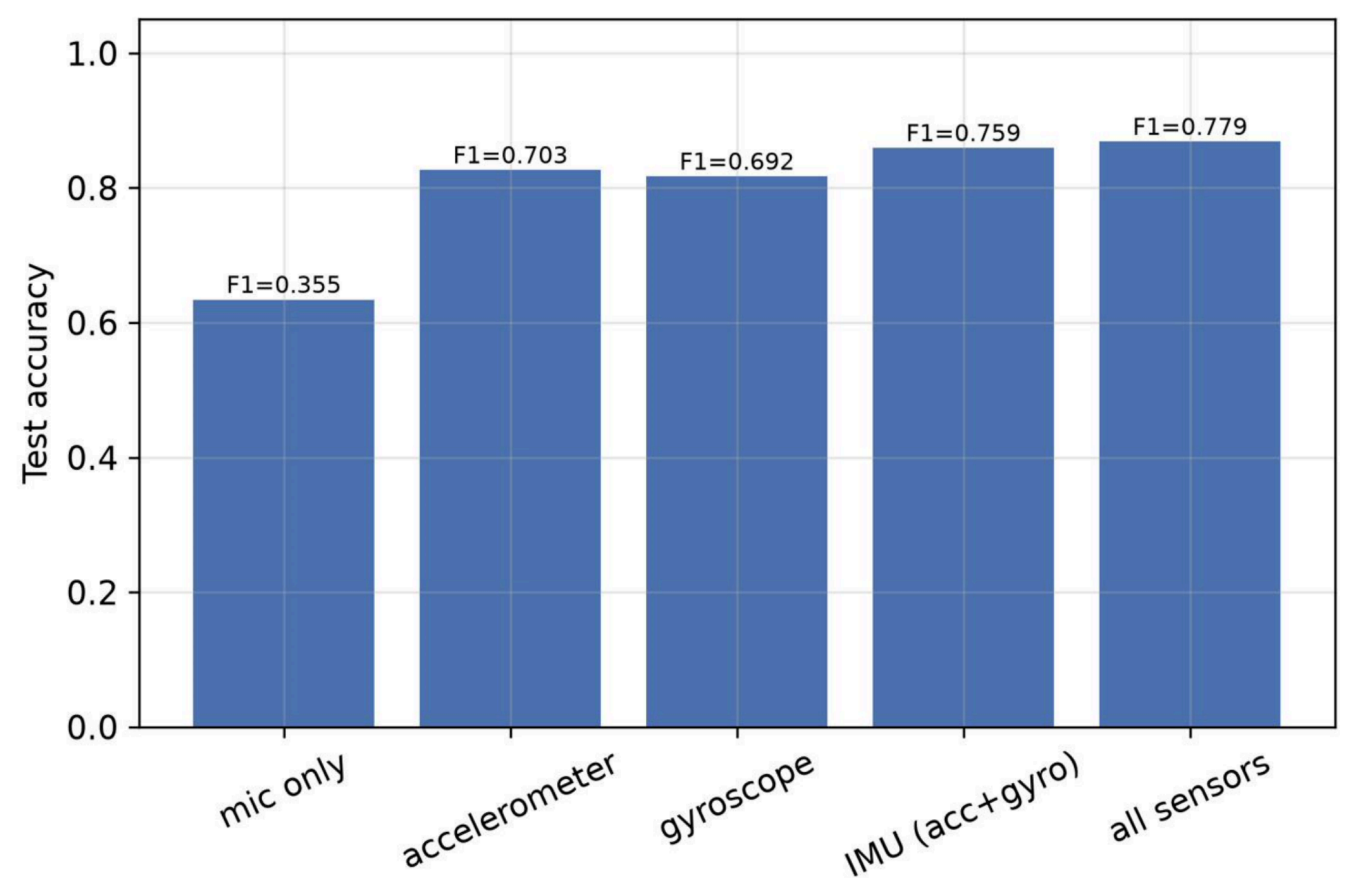
Sensor ablation results.

Table 12 and Figure 24 give the sensor ablation results. When only the microphone is used, the accuracy stays at the level of 0.6342, and the macro-F1 stays at 0.3546. When only the accelerometer is used, an accuracy of 0.8265 and a macro-F1 of 0.7034 are reached, and when only the gyroscope is used, the corresponding values are 0.8171 and 0.6921. The combination of the two inertial sensors gives an accuracy of 0.8593 and a macro-F1 of 0.7595, and with the addition of the microphone, the corresponding values rise to 0.8687 and 0.7787. The fact that the microphone is weak alone but useful as an addition shows that the acoustic channel carries an information component that the inertial channels miss.

This result shows an important difference from the ablation findings of our previous study. There, the removal of the acoustic and thermal channels led to the largest loss, and the inertial channels were described as complementary [37]. The reversal here can be linked to two reasons. The first one is that the temperature is no longer a model input, and this change removes the leakage based on the session identity. The second is that, with the split moved to the file level, the background noise specific to the recording session in the acoustic channel is no longer a discriminative clue.

**Figure 25:**
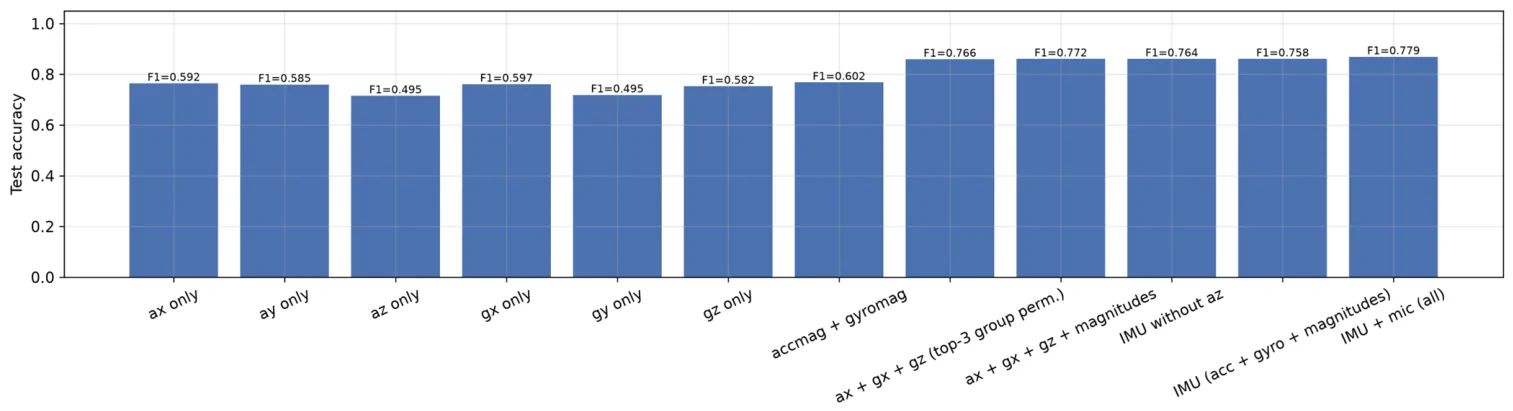
Inertial axis subset ablation results.

It should be noted that the sensor ablation compares the sensors at the level of the whole accelerometer and the whole gyroscope, and it does not search for the smallest subset of the inertial axes that can separate the fault classes. The group permutation results in Table 9 give a first indication for such a search. Accordingly, the Acceleration X, Gyro X and Gyro Z axes carry the largest contributions with accuracy drops of 0.0892, 0.0772 and 0.0755, while the Acceleration Z axis carries the smallest contribution with 0.0332. It is thought that this ordering is related to the orientation of the sensor with respect to the plane of rotation, in which the imbalance forcing acts. For this reason, a subset search has been carried out with the same reference classifier and the same split, and the results are given in Table 13 and Figure 25. Accordingly, a single axis reaches a macro-F1 between 0.4948 and 0.5969, and the Acceleration Z and Gyro Y axes give the lowest values with 0.4948, which is consistent with their low group permutation contribution. The two vector magnitudes alone reach 0.6022. The three-axis subset formed by Acceleration X, Gyro X and Gyro Z reaches an accuracy of 0.8593 and a macro-F1 of 0.7661 with 101 inputs, and the addition of the two magnitudes raises the macro-F1 to 0.7718 with 167 inputs. These values are higher than the value of 0.7580 obtained with all six axes and the two magnitudes with 266 inputs, and they stay only 0.0126 and 0.0069 below the value of 0.7787 obtained with all sensors. This result shows that the fault classes can be separated with three inertial axes and that the remaining axes carry mostly redundant information. It is thought that a signal representation which exploits the rotating nature of the response would reduce the number of channels further, but this requires an angular reference and is stated as a separate study in Section 4.

### 3.8. Frequency Representation Ablation

**Figure 26:**
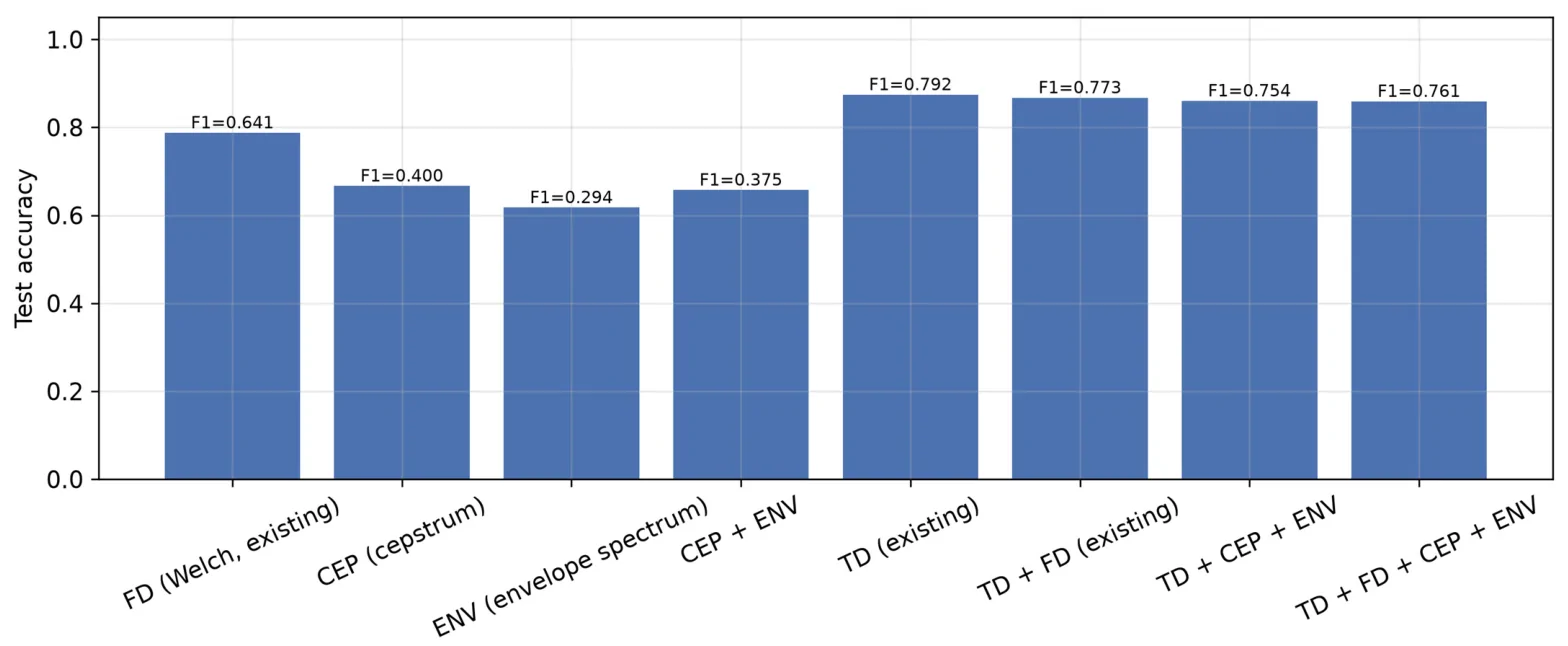
Frequency representation ablation results.

In order to answer the question of whether a representation that considers the rotating nature of the source gives more discriminative information than the Welch-based features at the present sampling rate, a cepstral and an envelope spectrum feature set have been computed on the same windows and compared with the existing sets by using the reference classifier and the same split. The real cepstrum of each channel has been computed from the logarithm of the Hann-windowed spectrum with 128 points, and the first eight cepstral coefficients, the frequency and the amplitude of the cepstral peak between two samples and 0.5 s and the cepstral energy have been taken as features, which gives 11 features per channel and 99 features for the nine channels. The envelope spectrum has been obtained from the Welch estimate of the Hilbert envelope of each channel, and the dominant envelope frequency and three band ratios have been taken, which gives 36 features. The two operating inputs have been added to every set. Table 14 and Figure 26 give the results. Accordingly, the cepstral set reaches a macro-F1 of 0.3997, and the envelope set reaches 0.2936, and both stay clearly below the Welch-based set with 0.6410. Adding the two sets to the time-domain set lowers the macro-F1 from 0.7922 to 0.7540, and adding them to the time and frequency domain set lowers it from 0.7728 to 0.7608. This result is consistent with the argument in Section 2.4. Because the shaft orders are folded into the band below 45.5 Hz and the window contains 91 samples, the cepstrum does not contain a resolved harmonic family, and the envelope spectrum does not contain an observable cyclic frequency. In other words, the failure of these representations on the present recordings is not evidence against cyclostationary analysis in general, but evidence that such an analysis needs an angular reference and a higher sampling rate, which is stated as a separate study in Section 4.

### 3.9. Operating Input Ablation

**Figure 27:**
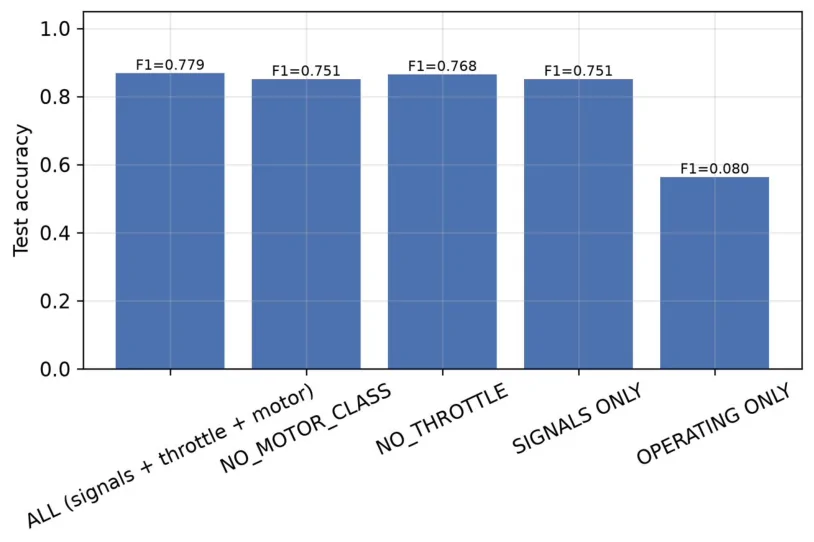
Operating input ablation results.

Table 15 and Figure 27 give the contribution of the operating inputs. The reference configuration where all the inputs are used gives an accuracy of 0.8687 and a macro-F1 of 0.7787. Removing the motor class lowers the macro-F1 value to 0.7508, and removing the throttle level brings it down to 0.7684. When both operating inputs are removed, the macro-F1 becomes 0.7515. The fact that these drops remain limited shows that the largest part of the performance comes from the vibration and acoustic signals.

The most critical row of this section is the configuration where only the operating inputs are used. With two inputs, the accuracy reaches a relatively high value of 0.5639, but the macro-F1 remains at 0.0801. Reading these two numbers together shows that the model predicts only the majority class with the throttle level and motor class information and cannot separate any fault type. Therefore the operating inputs provide context information but do not carry the classification decision alone. This finding is direct evidence that the operating inputs are not a source of leakage.

### 3.10. Cross Motor Generalization

**Figure 28:**
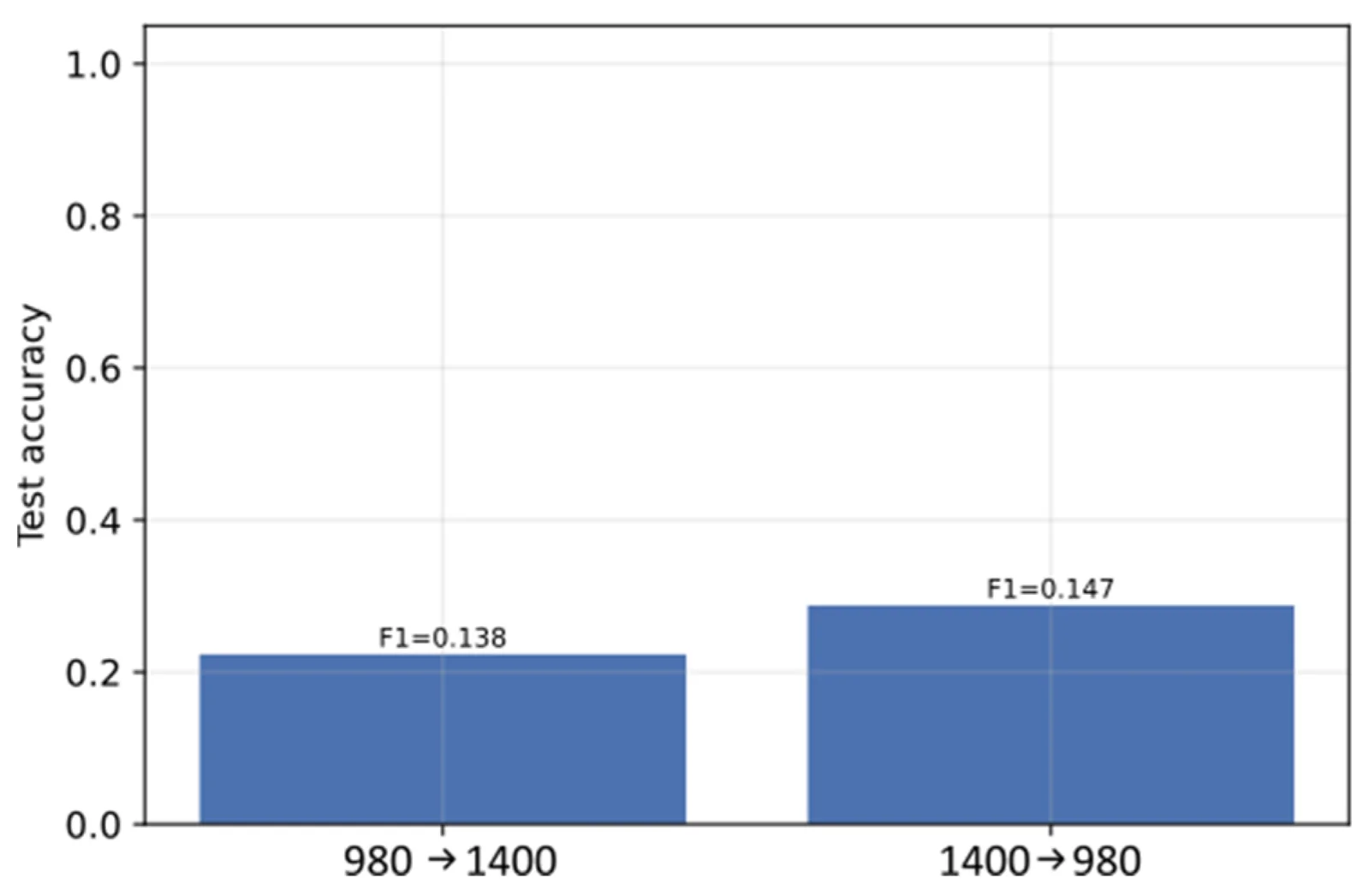
Cross-motor generalization results.

Table 16 and Figure 28 give the most challenging experiment. The model trained on the 6377 windows of the 980 kV motor obtains an accuracy of 0.2234 and a macro-F1 of 0.1380 on the 2113 windows of the 1400 kV motor. When the training is conducted in the reverse direction, the values are 0.2878 and 0.1468. In both directions, the performance is only at two or three times the level of a random guess in a problem with nine classes.

When this result is read together with the accuracy of about 0.87 obtained within the same split, it reveals an important limitation. The models do not learn the hardware-independent physical signature of the fault type but the vibration pattern produced by a particular motor and propeller combination. Because the rotation speed ranges and the mass moments of inertia of the two motors are different, the same throttle command produces different blade passing frequencies. The fact that the feature space does not contain a normalization that compensates this shift explains the drop. In terms of the application, this finding shows that a separate calibration dataset will be needed for each hardware configuration, or that domain adaptation methods have to be included in the pipeline.

The size of this drop deserves a more detailed discussion, because the generalization between operating conditions is a common problem of data-driven fault diagnosis and not a defect specific to this dataset. Three mechanisms explain the result. The first one is the shift of the marginal feature distribution. At the same throttle command, the 1400 kV motor rotates about 1.4 times faster than the 980 kV motor, and the amplitude of the imbalance forcing grows with the square of the rotation speed. Therefore, the mean, the root mean square and the energy features, which are at the top of the importance ranking in Section 3.6, move to a different range in the target motor. Tree ensembles partition the feature space with axis-aligned thresholds learned from the source range, and they cannot extrapolate beyond this range. The second one is the aliasing explained in Section 2.4. Because the shaft frequency is above the Nyquist frequency, a change in the rotation speed does not shift the spectral content in a monotonic way but folds it to a different position, so the band ratio features of the two motors are not related by a simple scaling. The third one is the direction of the error. The healthy class forms about 57 percent of the windows of both motors, so a model that falls back to the majority class would reach an accuracy near 0.57. The observed values of 0.2234 and 0.2878 are far below this level, which shows that the shifted features do not fall into the healthy region of the decision space but into the regions of the fault classes. In other words, the higher forcing of the faster motor is read by the model as a fault signature. It is thought that the slightly higher value in the 1400 to 980 direction comes from the fact that the source motor covers the higher amplitude range, so a part of the target range remains inside the learned thresholds.

This finding places the study in the same situation as the variable operating condition problem studied in the mechanical fault diagnosis literature, where domain adaptation, transfer learning and, recently, large language model-based adaptive methods are proposed to reduce the loss between the source and the target conditions [32,33,34]. The countermeasures that fit the present pipeline are the normalization of the amplitude features by the rotation speed, the use of order-based features obtained with an angular reference, the standardization of the feature vector per motor, and the alignment of the feature distributions of the two motors with domain adaptation methods before the classifier. It is also thought that a small calibration set collected from the target motor and used for fine tuning would recover a large part of the loss, because the fault mechanisms are the same and only the scale of the response changes. These extensions require new measurements and new training runs, so they are stated as a separate study in Section 4.

### 3.11. Statistical Verification

Table 17 presents the values obtained when the grouped split is repeated with five different seed values. The accuracy varies between 0.8499 and 0.8970, with a mean of 0.8738 and a standard deviation of 0.0179. The macro-F1 value fluctuates between 0.6965 and 0.7946, with a mean of 0.7592 and a standard deviation of 0.0347. The fact that the fluctuation observed for the macro-F1 is about twice that of the accuracy shows that the minority classes are sensitive to the small number of files that fall into the test partition. The values reported in the main text stay inside this range, which confirms that the results do not rely on a single random split.

Table 18 gives the result of testing the difference between the best representatives of the ensemble and deep branches with a paired McNemar test. In the test set, only the ensemble model decides correctly on 226 windows, while only the convolutional network decides correctly on 23 windows. The chi square statistic with continuity correction has been computed as 163.87, and the corresponding *p* value has been found far below the limit of 0.001. Therefore, the advantage of the ensemble branch is not a random fluctuation. In addition, the presence of a reverse contribution of 23 windows suggests that the convolutional network is not completely unnecessary and that a combination strategy could provide a small gain in the future.

### 3.12. Deployment Cost

Table 19 presents the disk size, the number of parameters and the inference latency per window of each model. Three different operating points appear in the table. The convolutional networks are the lightest options with sizes between 0.12 and 0.34 MB, and MobileNetTF provides a latency of 0.0708 ms per window with 26,553 parameters. The extra trees and the random forest are the largest single models with sizes of 167 and 184 MB, but their latencies stay around 0.12 ms. The stacked generalization and the soft voting are not suitable for embedded deployment with a size of 767 MB.

When this table is read together with the grid results, it produces a practical recommendation. The memory requirement of the stacked generalization pair that gives the best macro-F1 value is too much for a typical flight computer. In contrast, the extra trees pair that works with the TOPK_XAI set needs only 52 inputs and a single model with a macro-F1 value of 0.7905. It is considered that the memory footprint of this configuration can be clearly reduced by decreasing the number of trees or by applying pruning.

### 3.13. Comparison with the Existing Studies

Table 20 compares this study with representative works in the literature. A direct comparison of the reported accuracy values would be misleading, because the numbers of classes, the data sources and, most importantly, the split protocols are different. For this reason, a separate column has been added to the table for the split protocol. In most of the examined studies the split protocol is either not stated or applied randomly at the sample level. In a design that works with overlapping windows, this choice allows parts of the same raw recording to be present both in training and in testing.

The most meaningful row of the comparison is the row built with our previous study that uses the same experimental setup [37]. There, an accuracy of 97.2 percent was reported with a random stratified split at the window level. On the same physical setup, with a dataset extended to cover two motor classes and 16 throttle levels and with a split that preserves the file integrity, the accuracy obtained is at the level of 88.16 percent. This drop of about nine points is not a loss of performance but the measurement of the realistic generalization performance. In addition, this study reports the per class metrics, the leakage audit, the explainability analyses and the cross-motor generalization together, which provides a more complete framework in terms of comparability.

## 4. Conclusions

In this study, propeller fault classification has been addressed on a multisensor dataset that consists of 1735 measurement files collected under two different motor classes and 16 throttle levels. Under a stratified 80/10/10 split that treats the physical measurement file as indivisible, 33 combinations formed by matching four scalar feature sets and three time–frequency representations with six ensemble methods and three convolutional networks have been evaluated in a single grid.

The highest macro-F1 value of 0.8027 and an accuracy of 0.8816 have been obtained with the stacked generalization model trained on the XAI-ranked subset of 52 inputs. The highest accuracy of 0.8839 belongs to the extra trees model on the time-domain set. Every method in the ensemble branch has passed every architecture in the compact convolutional branch tested in this study, and this difference has been confirmed with the McNemar test. The repeated split experiment has revealed that the accuracy has a mean of 0.8738 with a standard deviation of 0.0179 and that the macro-F1 value has a mean of 0.7592 with a standard deviation of 0.0347. When the same predictions are reduced to a healthy and faulty setting, the accuracy rises to 0.9156, and the macro-F1 rises to 0.9119, which shows that the difficulty comes from the fault type and not from the detection of the fault.

The explainability analyses have shown that the time domain statistics carry about three times more information than the Welch-based frequency-domain features used in this study and that the Acceleration X axis and the gyroscope axes give the highest contribution. The sensor ablation has revealed that the inertial measurement unit alone provides a macro-F1 of 0.7595 and that this value rises to 0.7787 with the addition of the microphone. The inertial axis subset ablation has shown that three axes, namely Acceleration X, Gyro X and Gyro Z, reach a macro-F1 of 0.7661 with 101 inputs, and the frequency representation ablation has shown that cepstral and envelope features stay below the Welch-based features at the present sampling rate. The stationarity audit has shown that the mean is stable in more than 94 percent of the inertial windows. The operating input ablation has confirmed that the throttle level and the motor class give a macro-F1 of only 0.0801 and are therefore not a source of leakage. The cross-motor experiment has shown that the models depend strongly on the motor class and that the transfer between hardware is a research problem on its own.

The study has the following limitations. First, all the measurements have been collected on a test bench in a controlled environment, so the effects of the airframe structure, the flight-induced airflow, the wind and the control loop are not present in the data, and the results have not been validated under actual flight conditions. Second, the dataset is imbalanced, and although the class balancing has been applied only on the training partition, the minority classes are represented by 23 to 65 windows in the test partition, which is the reason for the higher variance of the macro-F1 value across the seeds. Third, the sampling rate of 90.9 Hz is below the shaft frequency of the propeller, and no angular reference has been recorded, so the shaft orders cannot be resolved, and the frequency domain findings are valid only for the Welch-based features used here and for the cepstral and envelope features tested in Section 3.8. Fourth, the deep branch is limited to three compact convolutional networks on maps of 16 by 16 cells, so the comparison does not cover deep learning in general. Fifth, the dataset covers two motor classes and one propeller geometry, so the cross-motor result cannot be generalized to other hardware without new measurements. Sixth, the damages have been produced artificially with repeatable methods, and naturally occurring wear may show a different signature.

These findings underline that the high performance values reported in the fault diagnosis literature have to be read together with the split protocol. The additional analyses that follow from these limitations cannot be carried out within the scope of the present study, because the recordings contain neither an angular reference nor a sampling rate that resolves the shaft orders, and the remaining ones require new measurement and training campaigns. For this reason, they are planned as a separate study. In that study, it is planned to record the rotation speed with a tachometer at a higher sampling rate, to repeat the cepstral and envelope analysis of Section 3.8 with order tracked and cyclostationary representations, and to verify the three-axis inertial subset of Section 3.7 on the new recordings. It is also planned to train larger deep architectures on higher-resolution maps and on the raw signal, to examine domain adaptation and large language model-based adaptive methods between motor classes, and to repeat the measurements under flight conditions. In addition, the tuning of the decision threshold per class according to the cost of a missed fault and the combination of the ensemble and convolutional branches will be investigated.

## Figures and Tables

**Table 1:** Basic principles, assumptions, advantages and limitations of the propeller fault diagnosis approaches in the literature.

Group	Basic Principle	Main Assumption	Advantages	Limitations	Refs.
Model-based	Residual between the measured response and a dynamic model of the rotor	An accurate model of the rotor dynamics is available	Physical interpretation, very few training data	Sensitive to model mismatch and parameter drift	[7,12]
Signal-based, vibration indicators	Imbalance, amplitude or frequency indicators computed from inertial data	The rotor forcing is observable in the sensor band, and the threshold is platform specific	Low computational load, on-board sensor, real time	Mostly binary decision, threshold retuning for each platform	[10,13,14,15]
Signal-based, acoustic indicators	Harmonic or noise ratio indicators computed from the sound	The background noise is stationary	Contactless, sensitive to aerodynamic change	Environment-dependent, weak when used alone	[16,26,27,28]
Data-driven, hand-designed features	Time and frequency statistics matched with support vector machines or tree ensembles	Training and test data come from the same distribution	Multiclass, low-cost, explainable	Feature design effort, hardware-specific thresholds	[18,19,20]
Data-driven, deep representation learning	Raw signal or time-frequency maps learned with convolutional, graph or transformer networks	A large number of independent samples are available	No feature design, high reported accuracy	Data-hungry, sensitive to sample level splits, black box	[17,21,22,23,29,30]
Data-driven, large language models	Signals converted to text or tokens and fed to a pretrained language model	The knowledge of the pretrained model transfers to the signal domain	Adaptivity across operating conditions, textual explanation	Hundreds of millions of parameters, not suitable for embedded devices	[32,33,34]
This study	Hand-designed and time-frequency representations matched with ensembles and compact networks in one grid	File-level independence, constant throttle inside a file	Leakage audit, explainability-driven feature subset, ablation and cross-motor tests	Test bench only, 90.9 Hz sampling rate, compact networks only	-

**Table 2:** Measurement files and 1 s windows per propeller condition.

Condition	Measurement Files	Windows	Motor Classes
Healthy	989	4831	980/1400
Notch long	130	634	980/1400
Notch short	92	450	980/1400
Scratch	81	402	980/1400
Single cut long	50	241	980/1400
Single cut short	86	428	980/1400
Two cut long	101	492	980/1400
Two cut short	106	523	980/1400
Warped	100	489	980/1400
Total	1735	8490	980/1400

**Table 3:** Partition audit of the file-atomic stratified split.

Partition	Win.	W. Ratio	Files	F. Ratio	HE	NL	NS	SC	SL	SS	TL	TS	WA
train	6787	0.799	1387	0.799	3866	504	360	324	195	338	392	415	393
valid.	850	0.100	174	0.100	484	65	45	39	23	45	50	53	46
test	853	0.100	174	0.100	481	65	45	39	23	45	50	55	50

**Table 4:** Composition of the scalar feature vector.

Domain	Features per Channel	Channels	Total
Time domain	17	9	153
Frequency domain	16	9	144
Operating inputs	-	-	2
Total	-	-	299

**Table 5:** Stationarity audit of 1964 windows from 400 randomly selected files. Share of the windows in which each test does not reject stationarity at the 5 percent level.

Channel	Variance (F Test)	Mean (*t* Test)	No Trend (Reverse arr.)	KPSS Level
mic	0.903	0.865	0.680	0.865
ax	0.830	0.960	0.792	0.936
ay	0.868	0.963	0.843	0.945
az	0.837	0.947	0.841	0.931
gx	0.852	0.965	0.918	0.948
gy	0.842	0.953	0.889	0.930
gz	0.861	0.972	0.959	0.959

**Table 6:** Test set performance of all models, sorted by macro-F1.

Model	Branch	Dataset	Inputs	Accuracy	Macro-F1	Macro Prec.	Macro Rec.	Log Loss	Time (s)
Extra Trees	ensemble	Time + frequency domain	299	0.8769	0.7937	0.8868	0.7533	0.5012	109.7
Random Forest	ensemble	Time + frequency domain	299	0.8675	0.7838	0.8714	0.7381	0.5690	20.4
Stacking	ensemble	Time + frequency domain	299	0.8710	0.7673	0.8302	0.7464	0.5698	906.8
LightGBM	ensemble	Time + frequency domain	299	0.8746	0.7662	0.8182	0.7522	0.6914	53.0
SoftVoting	ensemble	Time + frequency domain	299	0.8652	0.7544	0.7858	0.7529	0.4662	318.3
XGBoost	ensemble	Time + frequency domain	299	0.8605	0.7519	0.7933	0.7309	0.5120	40.9
HistGradientBoosting	ensemble	Time + frequency domain	299	0.8488	0.7316	0.7296	0.7515	0.5600	100.2
ResNetTF	deep	Time–frequency domain	3586	0.6213	0.4912	0.4934	0.5140	1.5240	22.1
CNN2D	deep	Time–frequency domain	3586	0.4525	0.3829	0.3916	0.4086	1.7763	21.5
MobileNetTF	deep	Time–frequency domain	3586	0.4338	0.3704	0.3743	0.4260	1.6972	17.5

**Table 7:** Per-class performance of the best model (Extra Trees, TD + FD).

Class	Precision	Recall	F1-Score	Support
healthy	0.8739	0.9938	0.9300	481
notchlong	0.8406	0.8923	0.8657	65
notchshort	0.9744	0.8444	0.9048	45
scratch	0.8378	0.7949	0.8158	39
singlecutlong	0.6923	0.7826	0.7347	23
singlecutshort	0.9615	0.5556	0.7042	45
twocutlong	0.8810	0.7400	0.8043	50
twocutshort	0.9200	0.8364	0.8762	55
warped	1.0000	0.3400	0.5075	50
macro avg	0.8868	0.7533	0.7937	853
weighted avg	0.8855	0.8769	0.8657	853

**Table 8:** Complete feature set by model grid on the test partition.

Branch	Feature Set	Model	Inputs	Accuracy	Macro-F1	Weighted F1	Macro AUC	Time (s)
deep	CWT	CNN2D	1794	0.4279	0.3407	0.4547	0.7917	17.8
deep	CWT	MobileNetTF	1794	0.2591	0.2525	0.2760	0.7500	15.5
deep	CWT	ResNetTF	1794	0.3294	0.3271	0.3635	0.7972	19.9
deep	STFT	CNN2D	1794	0.5627	0.4508	0.5814	0.8440	20.3
deep	STFT	MobileNetTF	1794	0.4795	0.4041	0.5128	0.8321	17.9
deep	STFT	ResNetTF	1794	0.5510	0.4058	0.5684	0.8370	20.0
deep	STFT + CWT	CNN2D	3586	0.4525	0.3829	0.4855	0.8102	19.4
deep	STFT + CWT	MobileNetTF	3586	0.4244	0.3623	0.4599	0.8253	17.7
deep	STFT + CWT	ResNetTF	3586	0.6213	0.4912	0.6358	0.8600	22.4
ensemble	FD	ExtraTrees	146	0.8019	0.6788	0.7936	0.9406	30.2
ensemble	FD	HistGradientBoosting	146	0.7749	0.6186	0.7690	0.9294	48.2
ensemble	FD	RandomForest	146	0.7819	0.6355	0.7715	0.9332	16.1
ensemble	FD	SoftVoting	146	0.7855	0.6305	0.7766	0.9361	109.0
ensemble	FD	Stacking	146	0.7866	0.6245	0.7679	0.9244	451.5
ensemble	FD	XGBoost	146	0.7726	0.6115	0.7662	0.9290	28.1
ensemble	TD	ExtraTrees	155	0.8839	0.8007	0.8737	0.9764	24.4
ensemble	TD	HistGradientBoosting	155	0.8675	0.7591	0.8628	0.9762	26.5
ensemble	TD	RandomForest	155	0.8722	0.7874	0.8612	0.9709	12.2
ensemble	TD	SoftVoting	155	0.8746	0.7817	0.8661	0.9763	75.9
ensemble	TD	Stacking	155	0.8710	0.7629	0.8633	0.9749	308.8
ensemble	TD	XGBoost	155	0.8640	0.7587	0.8583	0.9736	23.8
ensemble	TD + FD	ExtraTrees	299	0.8769	0.7937	0.8657	0.9787	69.3
ensemble	TD + FD	HistGradientBoosting	299	0.8488	0.7316	0.8488	0.9754	77.3
ensemble	TD + FD	RandomForest	299	0.8675	0.7838	0.8566	0.9721	19.5
ensemble	TD + FD	SoftVoting	299	0.8675	0.7648	0.8621	0.9771	197.0
ensemble	TD + FD	Stacking	299	0.8710	0.7730	0.8620	0.9752	673.2
ensemble	TD + FD	XGBoost	299	0.8605	0.7519	0.8539	0.9759	35.9
ensemble	TOPK_XAI	ExtraTrees	52	0.8769	0.7905	0.8663	0.9711	8.8
ensemble	TOPK_XAI	HistGradientBoosting	52	0.8664	0.7606	0.8640	0.9713	42.8
ensemble	TOPK_XAI	RandomForest	52	0.8640	0.7710	0.8571	0.9685	11.9
ensemble	TOPK_XAI	SoftVoting	52	0.8710	0.7839	0.8682	0.9728	68.7
ensemble	TOPK_XAI	Stacking	52	0.8816	0.8027	0.8750	0.9657	181.5
ensemble	TOPK_XAI	XGBoost	52	0.8417	0.7398	0.8422	0.9703	16.7

**Table 9:** Sensor group permutation importance for the best ensemble model.

Sensor Group	Accuracy Drop	Std
ax	0.0892	0.0065
gx	0.0772	0.0064
gz	0.0755	0.0037
mic	0.0473	0.0078
ay	0.0457	0.0050
operating_inputs	0.0435	0.0044
gy	0.0355	0.0046
az	0.0332	0.0052

**Table 10:** Domain group permutation importance for the best ensemble model.

Domain Group	Accuracy Drop	Std
time_domain	0.4185	0.0049
frequency_domain	0.1447	0.0119
operating_inputs	0.0420	0.0056

**Table 11:** Integrated gradients top 7 channel importance for the best CNN.

Channel	Mean |IG|	Channel	Mean |IG|
gz_cwt	0.02043	gy_stft	0.01720
ax_stft	0.01904	mic_cwt	0.01689
gx_stft	0.01902	az_cwt	0.01684
gy_cwt	0.01861	ax_cwt	0.01629
gz_stft	0.01855	gx_cwt	0.01529
mic_stft	0.01797	ay_stft	0.01476
ay_cwt	0.01760	az_stft	0.01365

**Table 12:** Sensor ablation. Throttle and motor class inputs are retained in every variant.

Sensor Set	Inputs	Accuracy	Macro-F1
mic only	35	0.6342	0.3546
accelerometer	101	0.8265	0.7034
gyroscope	101	0.8171	0.6921
IMU (acc + gyro)	200	0.8593	0.7595
all sensors	299	0.8687	0.7787

**Table 13:** Inertial axis subset ablation with the fixed reference classifier.

Subset	Inputs	Accuracy	Macro-F1
ax only	35	0.7644	0.5918
ay only	35	0.7597	0.5854
az only	35	0.7151	0.4948
gx only	35	0.7608	0.5969
gy only	35	0.7186	0.4948
gz only	35	0.7526	0.5816
accmag + gyromag	68	0.7679	0.6022
ax + gx + gz	101	0.8593	0.7661
ax + gx + gz + magnitudes	167	0.8605	0.7718
IMU without az	233	0.8605	0.7644
IMU (acc + gyro + magnitudes)	266	0.8605	0.7580
IMU + mic (all)	299	0.8687	0.7787

**Table 14:** Frequency representation ablation with the fixed reference classifier. FD: Welch-based features, CEP: cepstral features, ENV: envelope spectrum features, TD: time-domain features.

Feature Set	Inputs	Accuracy	Macro-F1
FD (Welch)	146	0.7878	0.6410
CEP	101	0.6671	0.3997
ENV	38	0.6178	0.2936
CEP + ENV	137	0.6577	0.3754
TD	155	0.8746	0.7922
TD + FD	299	0.8675	0.7728
TD + CEP + ENV	290	0.8605	0.7540
TD + FD + CEP + ENV	434	0.8593	0.7608

**Table 15:** Operating input ablation with a fixed reference classifier.

Variant	Inputs	Accuracy	Macro-F1
ALL (signals + throttle + motor)	299	0.8687	0.7787
NO_MOTOR_CLASS	298	0.8511	0.7508
NO_THROTTLE	298	0.8652	0.7684
SIGNALS ONLY	297	0.8511	0.7515
OPERATING ONLY	2	0.5639	0.0801

**Table 16:** Cross-motor generalization. Training on one motor class and testing on the other.

Train Motor	Test Motor	Train Windows	Test Windows	Accuracy	Macro-F1
980	1400	6377	2113	0.2234	0.1380
1400	980	2113	6377	0.2878	0.1468

**Table 17:** Repeated grouped splits with five random seeds.

Seed	Accuracy	Macro-F1
0	0.8499	0.6965
1	0.8970	0.7858
2	0.8903	0.7946
3	0.8731	0.7673
4	0.8588	0.7521
mean	0.8738	0.7592
std	0.0179	0.0347

**Table 18:** McNemar test between the best ensemble model and the best CNN.

Model A	Model B	Only A Correct	Only B Correct	Chi Square	*p* Value
best ensemble	ResNetTF	226	23	163.87	<0.001

**Table 19:** Deployment cost. Model size, parameter count and inference latency per window.

Model	Branch	Size (MB)	Parameters	Latency (ms/Window)
CNN2D	deep	0.176	41,801	0.0944
ExtraTrees	ensemble	167.025	-	0.1175
HistGradientBoosting	ensemble	11.347	-	1.4408
LightGBM	ensemble	14.597	-	0.3596
MobileNetTF	deep	0.115	26,553	0.0708
RandomForest	ensemble	183.987	-	0.1159
ResNetTF	deep	0.340	82,665	0.1621
SoftVoting	ensemble	767.423	-	0.3680
Stacking	ensemble	767.427	-	0.3625
XGBoost	ensemble	11.717	-	0.3677

**Table 20:** Comparison of the present study with representative works in the literature.

Ref.	Data Source	Sensors	Classes	Method	Split Protocol	Accuracy (%)
[13,14]	Flight, three platforms	Accelerometer	2	Time-domain recursive correlation	Not stated	Qualitative detection
[17]	On-board flight audio	Microphone array	Propeller configurations	DNN, CNN, LSTM, Transformer encoder	Not stated	98.30
[21]	Marine thruster, pool and lake	Hall current and hydrophone	4	Two-signal merged CNN	Not stated	99.94/99.06
[18]	Hexarotor flight	IMU	Propeller fault	FIR filters and sparse classifiers	Not stated	93.37/98.21
[20]	Hover test bench	Three-axis accelerometer	Unbalance levels	ReliefF ranking with SVM and kNN	Not stated	98.85
[16]	Test bench, three thrust levels	Microphone	2	HNR features and Gaussian naive Bayes	Not stated	81.24
[22]	Quadrotor flight	Flight data	Minor faults	Deep residual shrinkage network	Not stated	Above 98
[5]	Pre-flight, contactless	FMCW radar	Multiple abnormalities	Deep RF sensing	Not stated	97.5
[19]	Ground and flight	MEMS accelerometer	Propeller and screw looseness	FFT band features with KNN, DT, NN	Not stated	99.40
[37]	Same bench, 980 kV	Microphone, IMU, temperature	9	STFT with CNN, VGG, ResNet, EfficientNet, MobileNetV2	Window-level random stratified 80/20	97.20
This study	Same bench, 980 and 1400 kV, 1735 files	Microphone and six IMU axes	9	33 features per channel and STFT/CWT tensors, 7 ensemble and 3 CNN	File atomic stratified 80/10/10 with leakage audit	88.16 (macro-F1 0.803)

## Data Availability

The measurement dataset and the analysis code produced in this study will be shared in a publicly available repository after the paper is accepted for publication. The repository link will be sent to the editorial office before publication.

## Abbreviations

The following abbreviations are used in this manuscript:

CWT	Continuous wavelet transform
ESC	Electronic speed controller
FFT	Fast Fourier transform
IG	Integrated gradients
IMU	Inertial measurement unit
SHAP	Shapley additive explanations
STFT	Short-time Fourier transform
t-SNE	t distributed stochastic neighbor embedding
UAV	Unmanned aerial vehicle
XAI	Explainable artificial intelligence
